# Alphavirus Restriction by IFITM Proteins

**DOI:** 10.1111/tra.12416

**Published:** 2016-06-24

**Authors:** Stuart Weston, Stephanie Czieso, Ian J. White, Sarah E. Smith, Rachael S. Wash, Carmen Diaz‐Soria, Paul Kellam, Mark Marsh

**Affiliations:** ^1^ MRC Laboratory for Molecular Cell BiologyUniversity College LondonGower StreetLondonWC1E 6BTUK; ^2^ Wellcome Trust Sanger InstituteWellcome Trust Genome CampusHinxtonCB10 1SAUK; ^3^ Division of Infection and ImmunityUniversity College LondonGower StreetLondonWC1E 6BTUK

**Keywords:** alphavirus, IFITM, interferon inducible transmembrane protein, restriction factor, Semliki Forest virus, SFV, virus entry, virus–host interaction

## Abstract

Interferon inducible transmembrane proteins (IFITMs) are broad‐spectrum antiviral factors. In cell culture the entry of many enveloped viruses, including orthomyxo‐, flavi‐, and filoviruses, is inhibited by IFITMs, though the mechanism(s) involved remain unclear and may vary between viruses. We demonstrate that Sindbis and Semliki Forest virus (SFV), which both use endocytosis and acid‐induced membrane fusion in early endosomes to infect cells, are restricted by the early endosomal IFITM3. The late endosomal IFITM2 is less restrictive and the plasma membrane IFITM1 does not inhibit normal infection by either virus. IFITM3 inhibits release of the SFV capsid into the cytosol, without inhibiting binding, internalization, trafficking to endosomes or low pH‐induced conformational changes in the envelope glycoprotein. Infection by SFV fusion at the cell surface was inhibited by IFITM1, but was equally inhibited by IFITM3. Furthermore, an IFITM3 mutant (Y20A) that is localized to the plasma membrane inhibited infection by cell surface fusion more potently than IFITM1. Together, these results indicate that IFITMs, in particular IFITM3, can restrict alphavirus infection by inhibiting viral fusion with cellular membranes. That IFITM3 can restrict SFV infection by fusion at the cell surface equivalently to IFITM1 suggests that IFITM3 has greater antiviral potency against SFV.

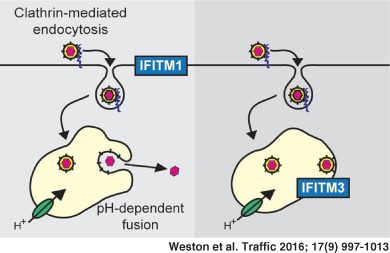

Human interferon inducible transmembrane proteins (IFITMs) are a family of five, 15–17 kDa membrane‐associated proteins, of which three (IFITM1, 2 and 3) appear to function as broad‐spectrum inhibitors of viral replication. Although detailed studies are lacking for most viruses, work on influenza A virus (IAV) has suggested that IFITM3, in particular, inhibits viral entry by interfering with endosomal, low pH‐induced fusion 1, 2, 3, 4. However, the precise molecular mechanism(s) for this inhibition remains unclear, and for some viruses alternative modes of action have been proposed 5, 6, 7.

Alphaviruses, especially Semliki Forest virus (SFV) and Sindbis virus (SINV), have been used extensively to study viral entry into cells 8, 9, 10, 11. These small (∼75 nm diameter), positive sense, single‐stranded RNA viruses were amongst the first to be shown to use clathrin‐mediated endocytosis and endosomal low pH‐dependent fusion to enter cells 8, 10. Despite the wealth of knowledge about their entry, IFITM‐mediated inhibition of alphavirus infection has not been analyzed in any great detail, although an overexpression screen has suggested IFITMs inhibit Chikungunya virus infection but had less affect on Venezuelan equine encephalitis virus 12. It has also been suggested that low pH‐induced fusion of SFV envelope glycoprotein expressing cells (fusion from within) can be inhibited by IFITM1 and IFITM3 3. Nevertheless, there is a view that alphavirus infection is not restricted by IFITMs 13, 14. Given the similarities, in terms of structure and mode of entry, between alphaviruses and flaviviruses, which are restricted by IFITMs 15, 16, 17, 18, we investigated whether SFV and SINV can also be restricted.

We show that normal infection by both SFV and SINV is restricted by IFITM3 and, to a lesser extent, by IFITM2, but not by IFITM1. The expression of IFITM3 does not affect SFV binding, internalization or entry into early endosomes, which also contain IFITM3. Moreover, the SFV E1 glycoprotein undergoes a characteristic low pH‐induced conformational change with similar kinetics in both IFITM3‐expressing and non‐expressing cells. However, release of the viral capsid protein into the cytosol is inhibited in IFITM3‐expressing cells.

SFV infection induced by low‐pH fusion with the cell surface was inhibited by the plasma membrane localized IFITM1, but was equally restricted by IFITM3, even though this protein is predominantly localized to intracellular compartment and is present at only low levels on the cell surface. Furthermore, a mutant of IFITM3, which is localized to the plasma membrane (Y20A), inhibited infection by plasma membrane fusion more potently than IFITM1, but did not inhibit the endosomal route of infection.

Together our results show that (i) alphaviruses are restricted by IFITMs, (ii) for SFV at least, IFITM3‐mediated restriction appears to affect viral fusion and cytosolic delivery of the viral capsid, and (iii) that IFITM1 and IFITM3 have different potencies for inhibition of SFV infection.

## Results

### IFITMs can restrict alphavirus infection

**Figure 1:**
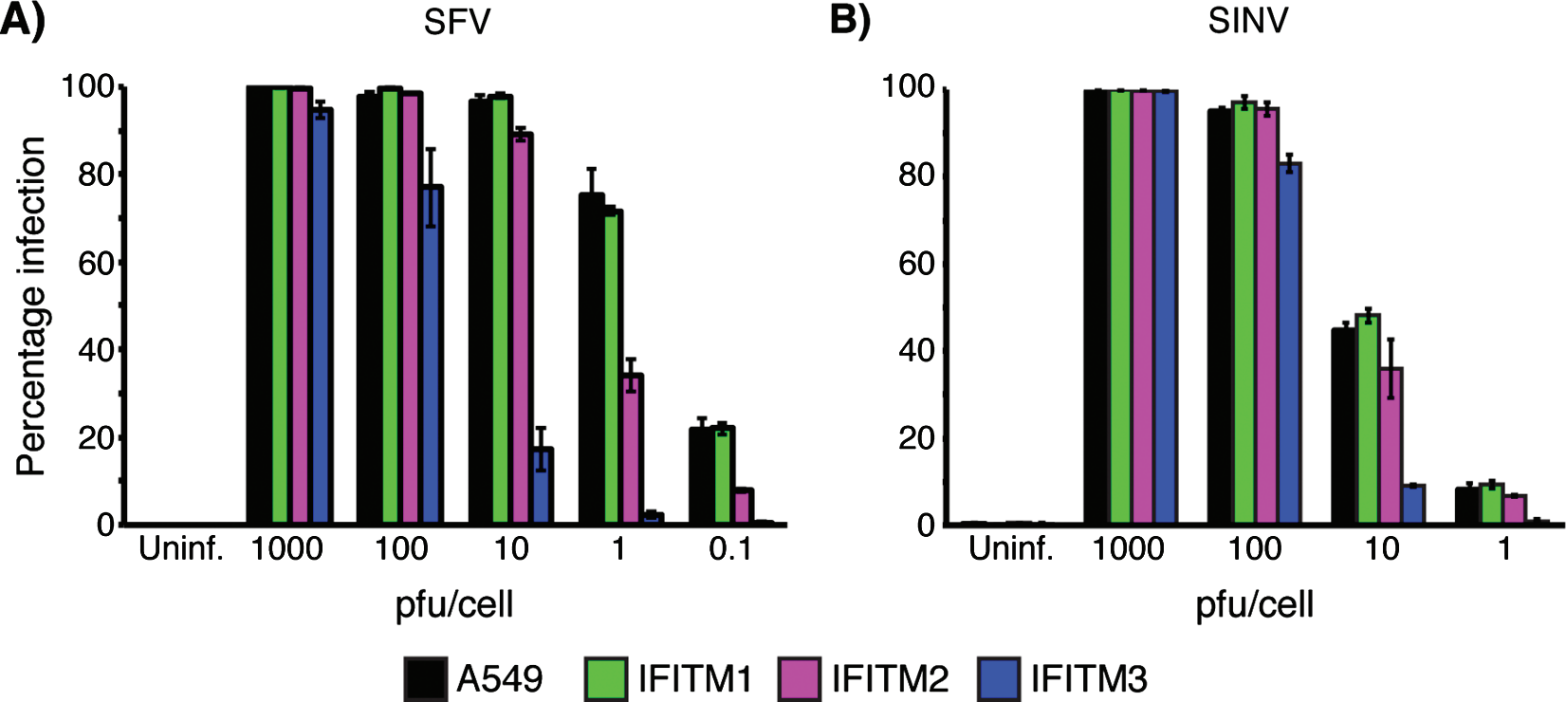
**Alphavirus infection is inhibited by IFITM proteins**. A549 cells stably expressing HA‐tagged IFITM1, 2 or 3 were infected with SFV (A) or SINV (B) at the indicated MOI (pfu/cell) for 5.5–6 h prior to fixation. Cells were then stained for the respective viral envelope proteins and the proportion of infected cells counted using an Opera microscope system. Although SINV infection of A549 cells was not as efficient as SFV, IFITM3‐HA expression inhibited infection by both viruses. IFITM2‐HA expression had some effect on SFV infection at lower MOI, but minimal effect on SINV infection. IFITM1‐HA had no effect on either virus. These data are from a representative experiment (n = 2–3) performed with triplicate wells in a 96 well plate. Each bar shows the mean infection percentage for the three wells and the standard deviation.

To investigate whether IFITMs can restrict infection by alphaviruses, we used A549 cells stably expressing human C‐terminally HA‐tagged IFITM1, 2 or 3 19, 20. Cells were infected with SFV or SINV at MOIs ranging from 0.1 to 1000 pfu/cell for 5.5–6 h, prior to immunolabelling for newly synthesized viral envelope glycoproteins (E1/E2) as a marker of infection. IFITM3‐HA inhibited infection by both viruses (Figure 1). At 1 pfu/cell, IFITM3‐HA expression inhibited SFV infection by ∼95%. Though requiring a higher MOI to see equivalent levels of infection, replication of SINV was also inhibited by IFITM3‐HA. For both viruses, IFITM3‐HA restriction was less efficient at higher MOIs (∼20% inhibition at 100 pfu/cell and <10% at 1000 pfu/cell for SFV, and ∼10% inhibition at 100 pfu/cell for SINV). IFITM2‐HA also inhibited SFV infection (∼50% at 1 pfu/cell) but had little activity against SINV. IFITM1‐HA had no activity against either virus.

These data indicate that IFITM3‐HA, and to a lesser extent IFITM2‐HA, can restrict infection of A549 cells by two different alphaviruses, and that the restriction can be saturated with higher levels of input virus.

### IFITM3 endosomal localization and expression levels impact antiviral activity

The A549 IFITM‐HA cells used in Figure 1 were produced through single cell cloning of lentivirally transduced cells. To further test IFITM3‐mediated inhibition, we used two additional sets of A549 stable cells produced using puromycin selection. One set (P1) was produced to stably express C‐terminally HA‐tagged IFITM1, 2 or 3, or an empty vector control. The other set (P2) included cells expressing IFITM3‐HA, IFITM3‐HA with a Y20A mutation and a GFP control. The Y20A mutation disrupts a Yxxϕ type endocytosis signal in the IFITM3 N‐terminal domain (NTD), causing accumulation of the protein at the plasma membrane (21, Figures S1 and 8B). IFITM3‐Y20A‐HA allowed us to investigate the importance of endosomal localization for antiviral activity (see Figures S1 and 3 for wild type IFITM3‐HA localization). These three sets of cells are denoted as: OS – the original set produced by single cell cloning (Figure 1), and P1 and P2 for the puromycin selected cells.

**Figure 2:**
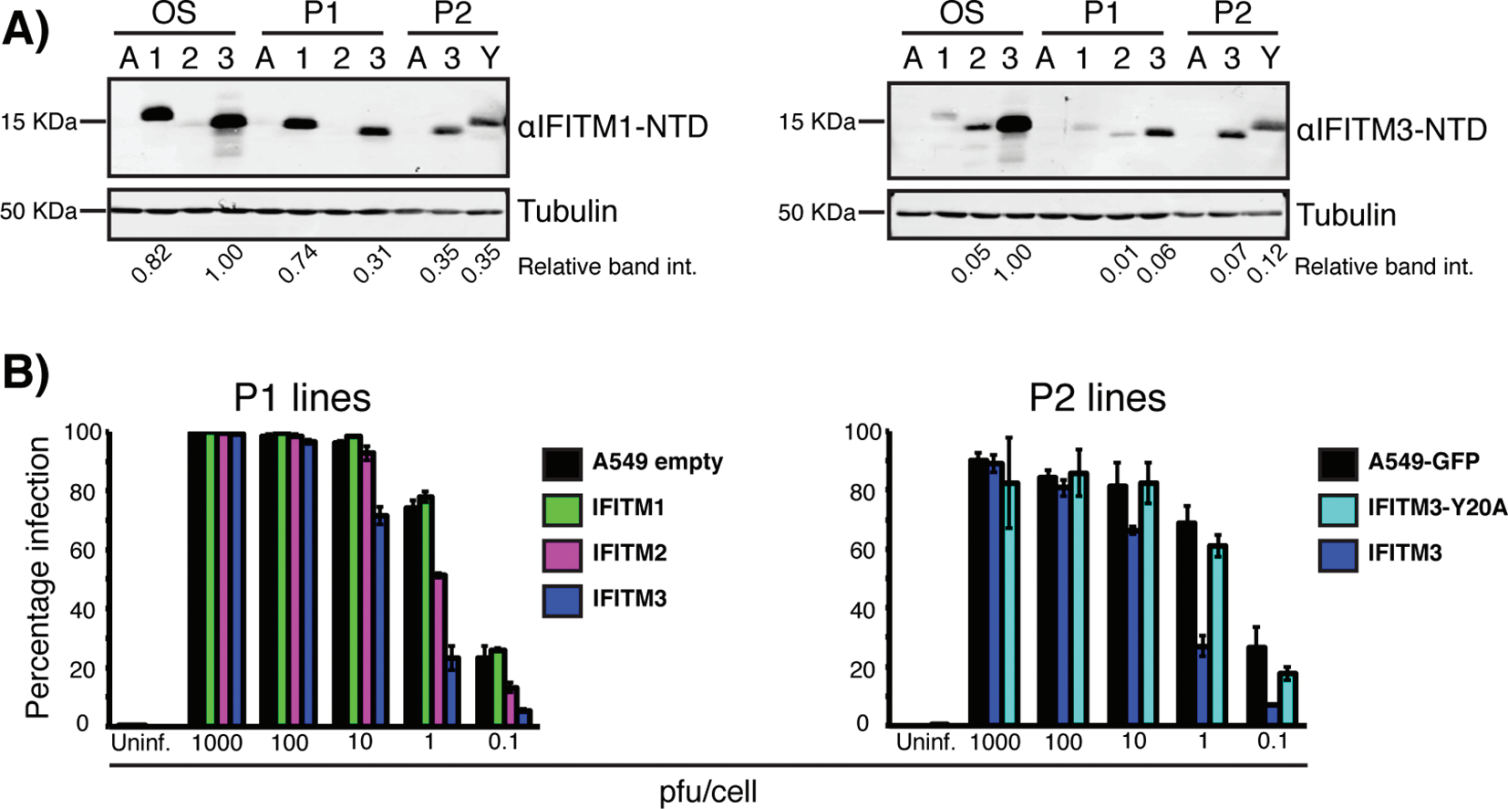
**IFITM3 expression levels affect SFV restriction**. Two sets of A549 cells stably expressing IFITM‐HAs (P1 and P2: See Materials and Methods) were analyzed for IFITM expression levels and SFV restriction. A) Western blotting was used to analyze the IFITM expression of all three sets of cells (OS = the original single cell clones used in Figure 1). A, 1, 2 and 3 denote A549, IFITM1, IFITM2 and IFITM3, respectively. Antibodies against the IFITM1‐NTD (which cross‐react with IFITM3) or IFITM3‐NTD (which cross‐react with IFITM2) were used to analyze IFITM expression. Tubulin was used as a loading control. Fluorescence intensity (int.) of the IFITM and tubulin bands was quantified using LiCOR Odyssey software. IFITM band intensities were normalized to tubulin loading and arbitrarily set relative to the highest band intensity on each blot (OS‐IFITM3‐HA in both cases). B) The P1 and P2 sets were infected with SFV across a range of MOIs and analyzed for infection by immunofluorescence, as in Figure 1. In line with lower IFITM expression, P1‐ and P2‐IFITM3‐HA show reduced levels of SFV restriction compared to OS‐IFITM3‐HA (Figure 1). These data are from a representative experiment (n = 2) performed with triplicate wells in a 96 well plate. Each bar shows the mean infection percentage for the three wells and the standard deviation.

**Figure 3:**
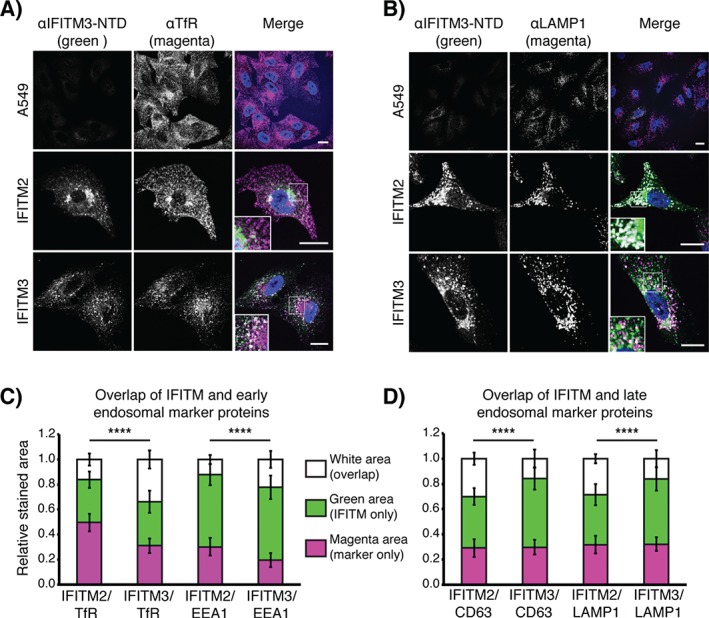
**IFITM2 and IFITM3 are found in endosomal compartments**. OS‐IFITM2‐HA and IFITM3‐HA cells were fixed, permeabilized and stained using an anti‐IFITM3‐NTD antibody, and markers of early and late endosomes and lysosomes. A) Example images of cells co‐stained for IFITM and transferrin receptor (TfR) and visualized with AF488 (IFITM – green) and AF647 (TfR – magenta) by confocal microscopy. Confocal sections are displayed. B) Example images of cells co‐stained for IFITM (AF488) and LAMP1 (AF647) and imaged as in (A). Nuclei were detected with Hoechst staining. Scale bars represent 15 µm. C and D) Colocalizations of IFITMs with early endosome markers, TfR and EEA1, or late endosome/lysosome markers, CD63 and LAMP1, were quantified. Data are from three independent experiments using between 18 and 21 fields of view collected using a 63× objective lens. Quantification of overlapping pixels and significance testing is described in Materials and Methods. IFITM3‐HA shows more overlap with TfR and EEA1 than IFITM2‐HA. Conversely, IFITM2‐HA shows greater overlap with CD63 and LAMP1 than IFITM3‐HA. There are pixels that contain just IFITM protein or just cellular marker, suggesting the IFITMs are not limited to single compartments. Bars show the mean pixel area and error bars are the standard deviation. ****p < 0.0001.

IFITM expression levels in all cells were analyzed by western blot (Figure 2A). Antibodies against the NTDs of IFITM1 or IFITM3 were used, as previously described 20. The OS cells had the highest IFITM expression. P1 and P2 cells had expression levels similar to each other. OS‐ and P1‐IFITM2‐HA had low expression, or poor detection (detected by cross‐reaction from the anti‐IFITM3‐NTD antibodies), possibly as a consequence of localization in more hydrolytic, late endosomal compartments (see Figure 3).

P1‐ and P2‐IFITM3‐HA expressing cells inhibited SFV in the range of 0.1 to 10 pfu/cell (Figure 2B), but restriction was reduced compared to that seen in OS‐IFITM3‐HA cells (compare Figures 2B with 1A). At 1 pfu/cell, OS‐IFITM3‐HA showed ∼95% inhibition while P1‐ and P2‐IFITM3‐HA cells showed ∼70% inhibition. The reduced levels of restriction correlate with the lower IFITM3‐HA expression. P1‐IFITM2‐HA cells showed some inhibition of SFV infection, though this was lower than OS‐IFITM2‐HA and all IFITM3‐HA cells. As with OS‐IFITM1‐HA, P1‐IFITM1‐HA did not restrict SFV infection. IFITM3‐Y20A‐HA, which localizes to the plasma membrane similarly to IFITM1‐HA (Figure S1 and 20), also did not inhibit SFV infection, suggesting that the endosomal localization of IFITM3 is essential for its anti‐SFV activity.

Previously, we noted that OS‐IFITM3‐HA restriction was less efficient at high viral input (Figure 1). This effect was also observed for the P1‐ and P2‐IFITM3‐HA cells. Thus, IFITM3‐mediated restriction can be saturated either by increasing the amount of virus or lowering the level of IFITM3‐HA expression.

Unless otherwise indicated, all subsequent work was performed with OS‐IFITM‐HA cells.

### IFITM3 preferentially localizes to early endosomes and IFITM2 to late endosomes

The majority of viruses restricted by IFITM3 are believed to fuse with late endosomes where some studies have suggested IFITM3 is localized 22, 23. SFV by contrast, has been shown to fuse in early endosomes at pH ≤ 6.2 8, 11. Since IFITM3‐HA more potently restricted SFV and SINV than IFITM2‐HA, and the endosomal localization of the protein is necessary for its antiviral activity, we investigated IFITM2‐HA and IFITM3‐HA localization. Cells were co‐immuno‐labeled for the IFITM proteins and markers of early and recycling endosomes [Early Endosomal Antigen 1 (EEA1) and transferrin receptor (TfR), respectively] or late endosomes and lysosomes [CD63 and lysosomal associated protein 1 (LAMP1)]. IFITM3‐HA showed more colocalization with TfR and EEA1 than IFITM2‐HA, while IFITM2‐HA showed more overlap with CD63 and LAMP1 than IFITM3‐HA (Figure 3). Image analysis showed that a significant amount of both IFITM2‐HA and IFITM3‐HA did not colocalize with each of the single endosome/lysosome markers (Figure 3C, D). Given that early endosomes are heterogeneous and neither EEA1 nor TfR mark the entire early endosome population, and IFITM2‐HA and IFITM3‐HA have somewhat overlapping distributions, we conclude that in fixed A549 cells IFITM3‐HA is associated more with early endocytic organelles and IFITM2‐HA more with later endocytic compartments. The higher levels of early endosomal localization of IFITM3‐HA may explain why this protein more effectively inhibited SFV and SINV infection than IFITM2‐HA, and the plasma membrane‐associated IFITM1‐HA and IFITM3‐Y20A‐HA proteins.

### IFITM3 expression does not block SFV binding or endocytosis

Having determined that IFITM3‐HA can inhibit alphavirus infection, and localizes to early endosomes, we hypothesized that the inhibition of viral protein production (the read out for Figures 1 and 2B) was due to inhibition of viral entry into cells. In order to infect a cell and replicate, alphaviruses need to deliver their RNA‐containing capsid into the cytosol. Initially the virus must bind to the cell surface, prior to internalization by clathrin‐mediated endocytosis (CME). Once in the endosomal system, the low pH environment triggers conformational changes in the envelope glycoproteins (E1/E2) to drive fusion of the viral and endosomal membranes and capsid release to the cytosol 24. We tested each of these aspects of the SFV entry pathway to determine the stage at which IFITM3 restricts infection.

**Figure 4:**
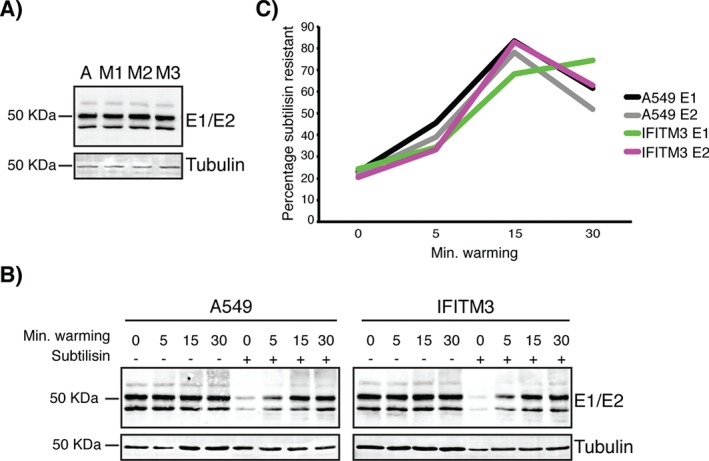
**SFV binding to and internalization into IFITM expressing cells** A) SFV (200 pfu/cell) was added to A549 cells or OS‐IFITM expressing cells (A, M1, M2, M3) at 4°C. After 1 h, the cells were washed and analyzed by western blotting for the viral envelope proteins. Tubulin was used as a loading control. The results indicate similar levels of binding to all four cell lines. B) SFV (200 pfu/cell) was allowed to bind to cells at 4°C for 1 h. Endocytic uptake of virus was promoted by warming to 37°C for 0, 5, 15 or 30 min. Cells were then treated with subtilisin at 4°C to remove surface‐bound virus, leaving only internalized virus associated with the cells. In each blot, controls of samples not treated with subtilisin were loaded to indicate the total cell‐associated virus. C) Quantification of E1 and E2 subtilisin resistant band intensity. 3–4 independent experiments were performed. Band intensities of E1 (bottom) and E2 (top) were determined and adjusted based on tubulin band intensity for each sample. A measure of total cell‐associated virus was determined by averaging band intensity for all untreated time points. The intensity of E1 or E2 at each treated time point was then set as a proportion of this total. Band intensities were averaged across experiments and plotted to display the percentage of subtilisin resistant E1 or E2 at each time point.

Initially, we investigated whether IFITM‐HA expression affected virus binding to the cell surface. Equal amounts of virus were added to IFITM‐HA expressing A549 cells for 1 h at 4 °C, and the amount of bound virus determined by western blotting whole cell lysates for E1/E2. Similar amounts of bound virus were detected in IFITM negative A549 cells and cells expressing each of the three IFITM‐HA proteins (Figure 4A).

Next, to investigate whether IFITM3‐HA affected SFV endocytosis, we measured virus uptake using a protease resistance assay 9. SFV was bound to the surface of cells as previously, and the cells then warmed to 37 °C for 5, 15 or 30 min to promote endocytosis. After each time point, the cells were placed on ice and treated with subtilisin 25. Samples were lysed and analyzed for E1/E2 by western blot. To quantify uptake, the band intensities of E1 (bottom) and E2 (top) were measured across multiple experiments. Although subtilisin failed to remove ∼22% of the surface virus, there was a clear increase in subtilisin resistant virus on warming the A549 cells to 37 °C (Figure 4B). This peaked at 15 min when > 75% of the virus was internalized (Figure 4C). Very similar uptake was seen on IFITM3‐HA cells. We conclude that IFITM3‐HA expression does not affect either binding, or internalization of SFV into A549 cells.

### Internalized SFV colocalizes with IFITM3

**Figure 5:**
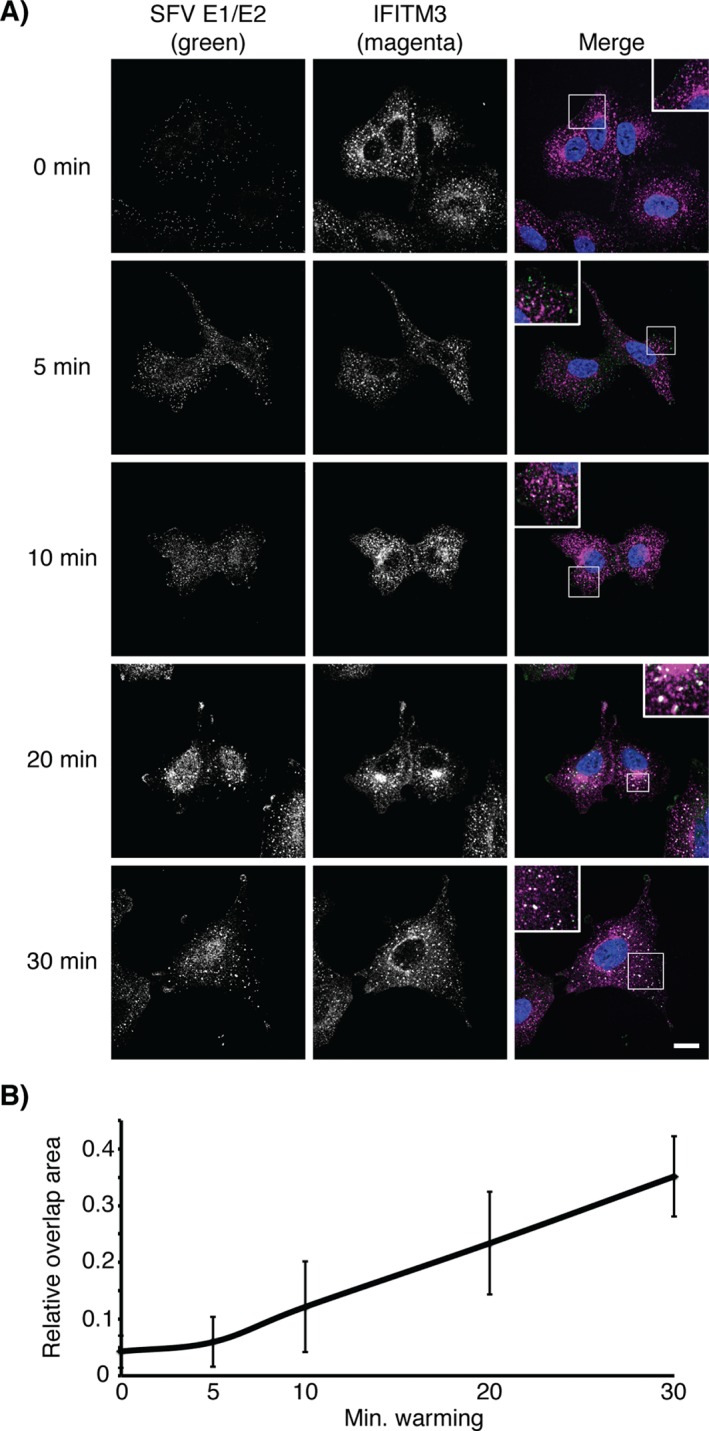
**Internalized SFV colocalizes with IFITM3**. A) SFV (50 pfu/cell) was bound to cells at 4°C for 1 h. After washing, the cells were warmed to 37°C for the indicated times, then fixed and labeled for SFV E1/E2 and OS‐IFITM3‐HA (via the HA‐tag) and visualized with AF488 (green, E1/E2) and AF647 (magenta, HA). Confocal sections are displayed. E1/E2 labelling can be seen as small puncta mainly around the cell periphery at 0 and 5 min, with little overlap of SFV E1/E2 and IFITM3‐HA. At later time points (10 min and onwards) larger and brighter puncta become visible closer to the nucleus, and overlap of SFV E1/E2 and IFITM3‐HA is seen, which increases with time. Many IFITM3‐HA positive, SFV negative puncta, can be seen suggesting that not all IFITM3‐HA containing endosomes receive virus. By contrast, many SFV positive puncta co‐label for IFITM3‐HA at 20 and 30 min. Nuclei were detected with Hoechst staining. Scale bar represents 15 µm. B) The overlap between green (SFV E1/E2) and magenta (HA) pixels was quantified (see Materials and Methods). A total of three independent experiments were performed, and six images taken at 63× magnification for each. The average ratio of the relative area of overlapping pixels (green and magenta) to green pixels from each experiment is plotted, with the standard deviation used for the error bars.

Since we observed that IFITM3‐HA is associated with early endosomes (Figure 3), we investigated whether internalized SFV entered IFITM3‐HA positive endosomes. As above, virus was bound and internalized into cells, which were then fixed and immuno‐labeled for E1/E2 and IFITM3‐HA and analyzed by confocal microscopy (Figure 5). As a control, these experiments were repeated in A549 cells that were stained for EEA1 instead of IFITM3‐HA (Figure S2). When kept at 4 °C (*t* = 0) or warmed for 5 min, virus particles were seen as faint puncta primarily around the cell edges, and there was little overlap with IFITM3‐HA or EEA1. After 10 min at 37 °C, and at later time points, SFV staining appeared as larger, increasingly bright, punctae. The increase in EEA1 puncta intensity seen in Figure S2 was also seen in mock‐infected cells (data not shown), suggesting this may be due to cooling and warming cells. A time‐dependent increase in the overlap between E1/E2 and IFITM3‐HA (Figure 5B) or EEA1 (Figure S3B) was detected over multiple experiments. These data suggest that endocytosed SFV was delivered to IFITM3‐HA positive endosomes.

These observations were further confirmed by electron microscopy (EM), which revealed that SFV particles were internalized by CME (Figure S3). Subsequently, SFV particles were detected in multivesicular bodies that could be co‐labeled for SFV and IFITM3‐HA (Figure S4), further demonstrating that virus particles were delivered into IFITM3‐HA containing endosomes.

To confirm that we were investigating time points relevant to infection, the kinetics of SFV capsid release in A549 cells were determined. SFV penetration of endosomes is low pH‐dependent. Thus, ionophores such as monensin, that rapidly dissipate cellular low pH gradients, can be used in time of addition experiments to determine when incoming virus has passed through the pH sensitive stage of entry 26. Virus particles were bound to A549 cells prior to warming to promote uptake, as previously. Monensin was added at times between 0 and 30 min. When added at 0 min, monensin nearly completely abolished infection (Figure S5). However, when added at later times, an increasing percentage of cells became infected. By 30 min, monensin addition had almost no inhibitory effect (Figure S5), suggesting the majority of infectious virus had penetrated the cells. Therefore, analysing SFV internalization within the first 30 min of warming is relevant to infection.

### SFV is exposed to acidic pH in IFITM3 expressing cells

**Figure 6:**
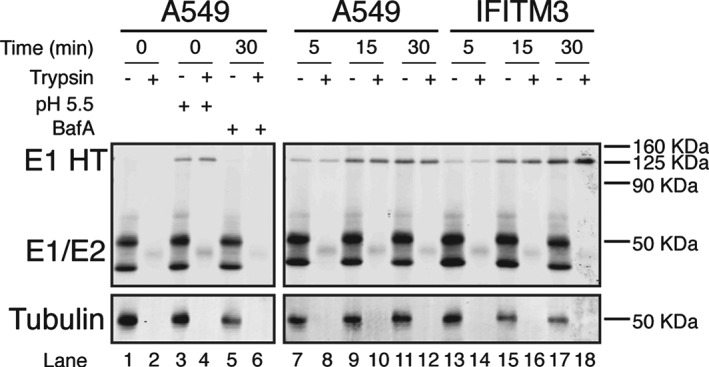
**SFV is exposed to acidic pH in IFITM3 expressing cells**. SFV (200 pfu/cell) was bound to A549 (lanes 1–12) or OS‐IFITM3‐HA expressing cells (lanes 13–18) at 4°C for 1 h. The cells were then washed and either kept at 4°C (lanes 1–4) or warmed to 37°C to allow virus uptake (lanes 5–18), as indicated. In lanes 3–4, cells with bound virus were briefly (3 min) treated with pH 5.5 medium at 37°C, or in lanes 5–6, were warmed to 37°C for 30 min in medium containing bafilomycin A1 (Baf A). Subsequently, all cells were placed on ice, lysed with 1% Triton X‐100 and half of the lysate treated with trypsin at 37°C as indicated. All samples were then analyzed by non‐reducing SDS‐PAGE and western blotting for SFV E1/E2 and tubulin as a loading control. Following a pH 5.5 pulse (lanes 3–4), or incubation at 37°C (lanes 7–18), a high MW band corresponding to the acid‐induced, trypsin‐resistant, E1 homotrimer (HT) was seen. This band was absent at 0 min (lanes 1–2) and in Baf A‐treated samples (lanes 5–6), indicating it is the low pH‐induced form of E1.

We next investigated whether the viral glycoproteins received the appropriate low pH trigger to become fusogenic in IFITM3‐HA expressing cells. The acid‐induced conformational changes in the E1/E2 complex generate a homotrimeric (HT) form of E1 that is resistant to trypsin digestion 27. Again, SFV was bound to cells and allowed to internalize for 0, 5, 15 or 30 min at 37 °C. As a positive control, virus particles bound to cells at 4 °C were treated with pH 5.5 medium for 3 min at 37 °C to activate the fusion protein directly at the cell surface. As a negative control, cells were pre‐treated with bafilomycin A1 (Baf A; a vacuolar ATPase inhibitor) for 15 min at 37 °C, prior to binding and internalization of virus particles in the presence of Baf A. After appropriate treatments, cells were lysed and the lysates treated with trypsin, or not, and the viral E1/E2 proteins analyzed by western blot. Samples were not heated prior to SDS‐PAGE as this can dissociate the E1 HT. With 0 min of internalization, the monomeric forms of E1 and E2 were seen with the expected molecular weights (MW) of ∼50 kDa (Figure 6, lane 1). After trypsin treatment of samples kept at 4°C, the E1/E2 bands were undetectable (lane 2). Transient low pH treatment of surface‐bound virus induced the formation of a high MW band that was resistant to digestion with trypsin (lanes 3 and 4), corresponding to the E1 HT. This trypsin‐resistant, high MW band was seen, with increasing intensity, when virus was allowed to internalize into A549 cells for 5, 15 or 30 min (lanes 7–12), but not when virus was internalized into Baf A treated cells (lanes 5 and 6). When SFV was internalized into IFITM3‐HA expressing cells the trypsin‐resistant, high MW band appeared with kinetics similar to those seen in IFITM‐negative A549 cells (lanes 13–18). We therefore conclude that IFITM3‐HA does not interfere with acidification of endosomes, virus trafficking to these compartments, or the conformational changes necessary for the viral glycoprotein to become fusogenic.

### IFITM3 expression inhibits release of SFV capsid to the cytosol

**Figure 7:**
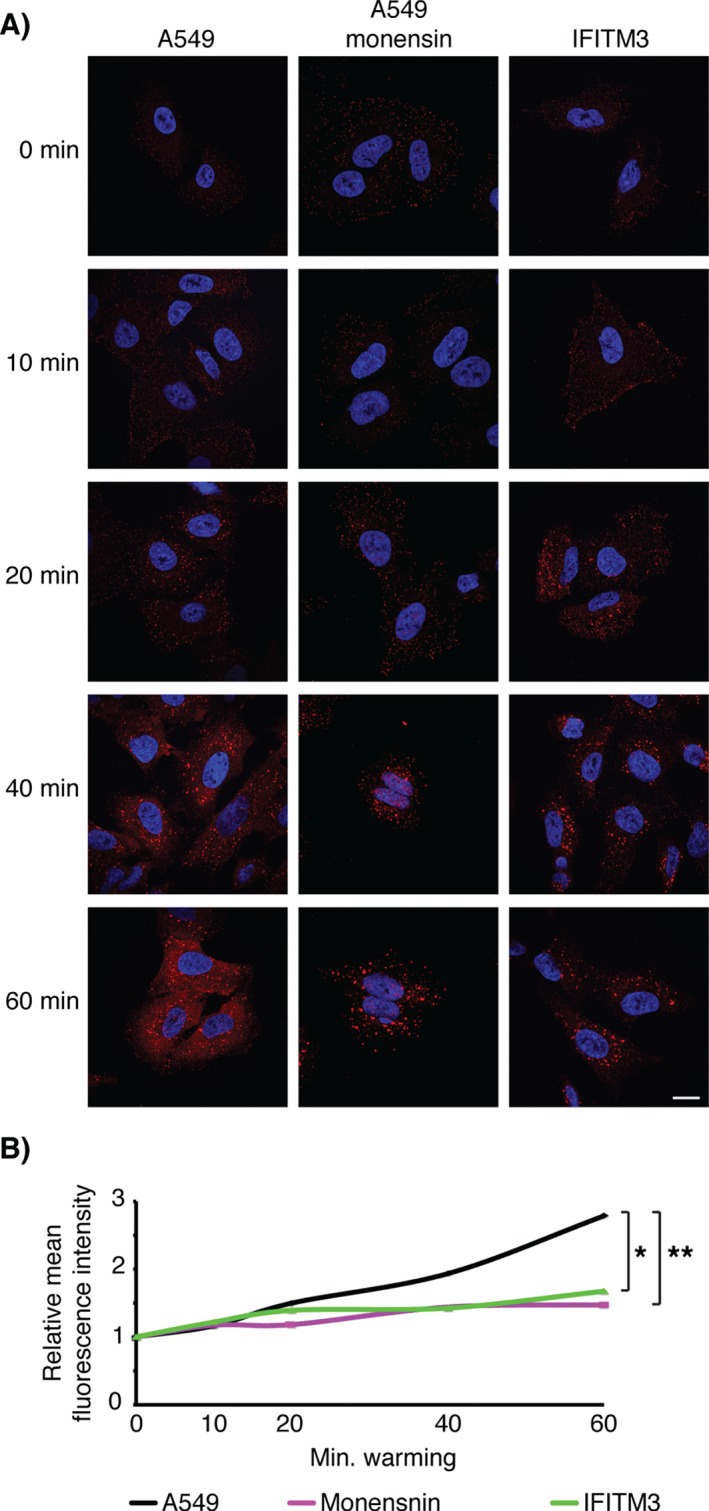
**IFITM3 expression inhibits SFV capsid release**. A) SFV (200 pfu/cell) was bound to cells for 1 h at 4°C prior to incubation at 37°C for the indicated times. At each time point the cells were fixed and permeabilized with 0.1% Triton‐X100 and labeled with serum against the SFV capsid, which was detected with AF594. Confocal sections are displayed. Following internalization, virus particles were detected in cells, indicated by the typical punctate association of virus with endosomes. By 40 min after warm‐up diffuse cytosolic fluorescence was seen in A549 cells, indicating release of the viral capsid protein to the cytosol. In A549 cells treated with 10 µm monensin, and in OS‐IFITM3‐HA expressing cells, the staining remains associated with puncta and the cytosolic staining was not observed even at 60 min after warm‐up. Nuclei were detected with Hoechst staining. Scale bar represents 15 µm. B) Quantification of cytosolic fluorescence and significance testing is described in Materials and Methods. The data are from three independent infections with 3–7 images taken at each condition, with a total of at least 60 cells analyzed per cell line, per condition. Data presented is the average fluorescence intensity, normalized to the background intensity at 0 min of warming. *p < 0.05, **p < 0.01.

The final step in the SFV entry pathway is release of the capsid into the cytosol. To determine whether IFITM3 affects this step, SFV particles were bound and internalized into cells, which were then analyzed by immunofluorescence staining for the SFV capsid protein. Between 0 and 20 min, in both A549 and IFITM3‐HA cells, punctae of virus were seen at the cell surface and subsequently within endosomes (Figure 7A), as observed by E1/E2 labelling (Figures 5 and S3). In general the labelling was weak, even after treatment with Triton, presumably because the capsid protein is poorly accessible when packed within virions. At 40 min in A549 cells, diffuse cytosolic fluorescence was seen, which was further increased at 60 min. This cytosolic fluorescence was not seen following SFV uptake into monensin‐treated A549 cells, or in IFITM3‐HA cells, suggesting the diffuse fluorescence is from viral capsid protein released into the cytosol. To develop a more quantitative analysis of this capsid release, images were analyzed over multiple experiments to determine the mean cytosolic fluorescence intensity in each cell. This quantification indicated that over time there is an increase in the cytosolic capsid protein‐associated fluorescence in A549 cells, that is not seen in monensin‐treated A549 or IFITM3‐HA cells (Figure 7B). In addition, in these latter cells the punctae of SFV staining appeared brighter at later times, suggesting that non‐fused virus particles accumulated within endosomes (Figure 7A). Together these observations indicate that IFITM3‐HA expression inhibits release of SFV capsid into the cytosol.

### IFITM1 and IFITM3 can inhibit SFV infection by fusion at the plasma membrane

Our data indicate that IFITM3 is primarily localized to early endosomes and can block SFV capsid release into the cytosol. We therefore investigated whether IFITM3‐mediated restriction could be bypassed by fusion of SFV at the cell surface, and whether plasma membrane localized IFITM1 20 or IFITM3‐Y20A (Figure S1) could restrict entry at this site. Cells were pre‐treated with Baf A for 15 min at 37°C to inhibit SFV entry via the endocytic route, prior to binding virus to the surface at 4°C for 1 h. The cells were then treated with pH 5.5 medium for 3 min at 37°C to induce fusion at the plasma membrane, prior to returning to pH 6.8 medium containing Baf A for 5.5–6 h at 37°C to allow production of E1/E2 proteins as a read out for infection. As controls, cells with bound virus were given a 3 min pH 6.8 pulse and treated with DMSO or Baf A.

**Figure 8:**
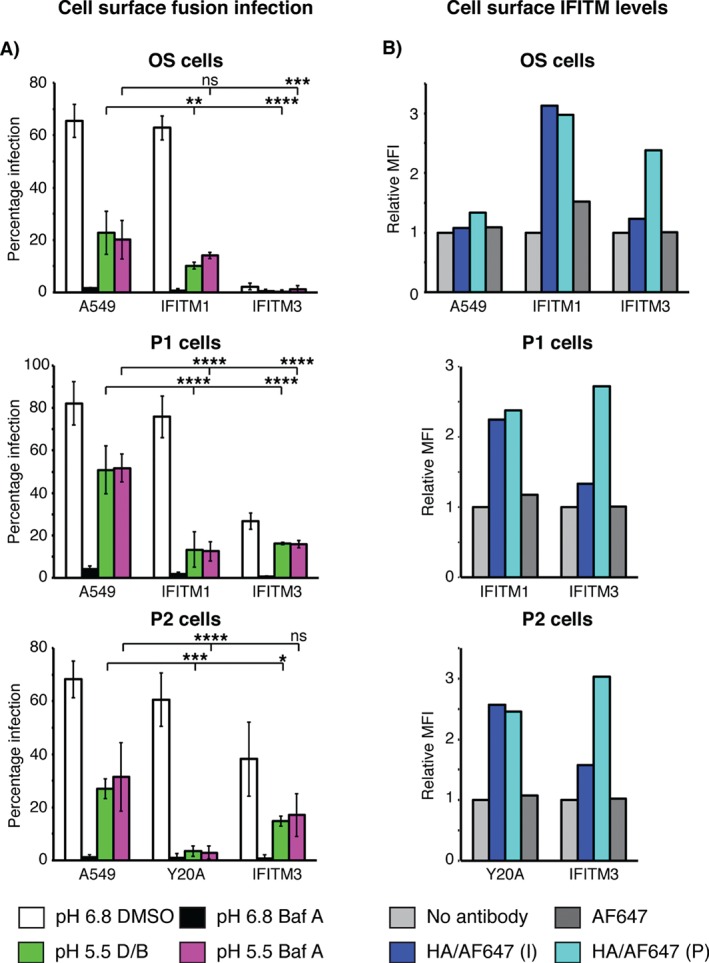
**IFITM1 and IFITM3 can inhibit SFV infection by fusion at the cell surface**. A) OS‐, P1‐ and P2‐IFITM‐HA cells were infected by SFV through plasma membrane fusion. Cells were pre‐treated with either DMSO or bafilomycin A1 (Baf A) prior to addition of SFV (5 pfu/cell) for surface binding at 4°C for 1 h (in the presence of DMSO or Baf A). Surface‐bound virions were then allowed to enter cells by endocytosis (pH 6.8) or fused directly at the cell surface (pH 5.5). pH 6.8 media containing DMSO or Baf A (matched to the pre‐treatment) was then added to the cells, which were incubated for 5.5–6 h at 37°C to allow infection. As a control for the Baf A pre‐treatment, cells were instead pre‐treated with DMSO, then incubated with Baf A containing media for the infection period (pH 5.5 D/B). Cells were fixed and infection determined by immunofluorescence microscopy. Baf A inhibited infection through the endocytic route (black bars compared to white bars). Low pH treatment resulted in bypass of Baf A inhibition, indicative of cell surface fusion (green and magenta bars). The bars indicate the mean infection percentages from three independent experiments (each containing triplicates for each sample) with standard deviation shown as the error bars. Statistical significance was determined using normalized infection values, comparing pH 5.5 DMSO/Baf A (green) or pH 5.5 Baf A (magenta) of A549 samples with IFITM samples, as detailed in the Materials and Methods section. *p < 0.05, **p < 0.01, ***p < 0.001, ****p < 0.0001. B) OS‐, P1‐ and P2‐IFITM‐HA cells were labeled with anti‐HA antibodies either intact (I) or following permeabilization (P), detected with AF647, and analyzed by flow cytometry to determine the relative levels of cell surface IFITM protein. As controls, cells were either incubated with no antibody or secondary antibody only. The mean fluorescence intensity (MFI), relative to the no antibody control for each cell line is displayed. Plots are representative of 3–4 independent experiments.

Through the normal endocytic route (pH 6.8 DMSO), between ∼65% and ∼80% of A549 cells (OS, P1 and P2), were infected with SFV (5 pfu/cell), and infection was inhibited by >90% with Baf A treatment (pH 6.8 Baf A; Figure 8A). When cells were pre‐incubated with Baf A and transiently treated with low pH medium, ∼20–50% of cells were infected (pH 5.5 Baf A; Figure 8A). The Baf A pre‐treatment step did not affect the result, since pre‐treatment with DMSO media, followed by Baf A media for the infection period (pH 5.5 DMSO/Baf A) showed similar results. This bypass of Baf A inhibition with pH 5.5 treatment is the level of infection by direct fusion at the plasma membrane. It appeared that the three A549 cells had different permissiveness to infection by plasma membrane fusion. The parental OS‐A549 cells were the least permissive to plasma membrane fusion; the P1‐A549 cells (stably transfected with an empty puromycin resistance vector) were the most permissive, while the P2‐A549 cells (stably transfected with a GFP construct) were in the middle. We speculate that the different manipulations of these cells may be responsible for the variability.

Virus entering through the endocytic route infected OS‐ and P1‐IFITM1‐HA cells, similarly to A549 controls, and Baf A inhibited this. When virus bound to the surface of both OS‐ and P1‐IFITM1‐HA cells was transiently treated with pH 5.5 medium, the infection percentage was consistently lower than that seen in similarly treated A549 cells (Figure 8A). In the OS set, the fact that A549 control cells were not easily infected by plasma membrane fusion meant that inhibition by IFITM1‐HA was modest. However, in the P1 set, the difference between infection by plasma membrane fusion of A549 and IFITM1‐HA cells was much greater, arguing that IFITM1‐HA can inhibit plasma membrane fusion to some extent.

Plasma membrane localized P2‐IFITM3‐Y20A‐HA did not restrict SFV infection through the endocytic route (Figures 8A and 2B). However, when surface‐bound virus was low pH treated, P2‐IFITM3‐Y20A‐HA inhibited infection by ∼95% (Figure 8A). This inhibition is greater than that seen for either OS‐ or P1‐IFITM1‐HA (Figure 8A), even though P2‐IFITM3‐Y20A‐HA expression levels were lower than those of IFITM1‐HA (Figure 2A).

Endocytic infection was low in all IFITM3‐HA cells, with OS‐IFITM3‐HA being the most restrictive (Figure 8A as in Figures 1 and 2B). Baf A reduced this low level infection to < 1%. Surprisingly, there was no increase in infection of these cells following pH 5.5 treatment of cell surface‐bound virus, and the infection was consistently lower than that seen in all A549 cells (Figure 8A). Indeed, OS‐IFITM3‐HA had the lowest infection percentages, even lower than either OS‐ or P1‐IFITM1‐HA. IFITM3‐HA also inhibited SFV infection via plasma membrane fusion in the P1‐ and P2‐ cells to a similar extent to that seen with IFITM1‐HA.

It was interesting to see such potent inhibition of cell surface fusion by IFITM3‐HA since immunofluorescence microscopy indicated that the majority of IFITM3‐HA is located in endosomal compartments (Figure 3) rather than at the cell surface. To confirm these observations, cells were stained either intact, or following permeabilization, and analyzed by flow cytometry to determine levels of each IFITM protein at the cell surface. Antibodies against the C‐terminal HA‐tag were used as we, and others, have shown the IFITM proteins to have a type‐II transmembrane topology with the C‐terminal domain facing the extracellular space 20, 28, 29. The mean fluorescence intensity (MFI) of labeled OS‐IFITM1‐HA, P1‐IFITM1‐HA and P2‐IFITM3‐Y20A‐HA was essentially equivalent between intact and permeabilized cells, suggesting the HA‐tag is accessible at the cell surface (Figure 8B). Conversely, for all three IFITM3‐HA cases, significant labelling was only detected when cells were permeabilized, suggesting the majority of IFITM3‐HA is in intracellular pools (Figure 8B). The MFI of intact IFITM3‐HA cells was slightly above background levels, suggesting some surface IFITM3‐HA (as we have previously observed 20),

Overall, despite low levels of cell surface expression (Figure 8B), IFITM3‐HA inhibited SFV infection by fusion at the plasma membrane to a similar or greater extent than IFITM1‐HA (Figure 8A). Presumably IFITM3‐HA that transiently transits the cell surface *en route* to endosomes is responsible for this inhibition 20, 21, 30. From this, we conclude that IFITM3‐HA is a more potent inhibitor of SFV fusion than IFITM1‐HA.

## Discussion

IFITM proteins restrict the replication of a wide range of enveloped viruses, and at least one non‐enveloped virus 22, 23. However, to date there is little evidence that IFITMs restrict alphavirus infection. Here we show that IFITM3, and to a lesser extent IFITM2, can inhibit the replication of two alphaviruses in A549 cells. Using SFV, we show that virus binding, endocytosis, delivery to endosomes and exposure to acidic pH are unaffected by IFITM3 expression. The viral envelope proteins undergo an acid‐dependent conformational change with similar kinetics in IFITM3 expressing and non‐expressing cells. However, the viral capsid protein does not appear in the cytosol of IFITM3 expressing cells. These results indicate that IFITM3 restricts SFV infection by inhibiting endosomal fusion and/or uncoating of the viral capsid.

To date the broad antiviral activity of IFITMs has been largely seen through the use of retrovirus‐based pseudotype reporter systems 22. However, for influenza A virus (IAV) the inhibitory activity of IFITM3 has been better characterized and appears to occur following virus endocytosis and delivery to endosomes, but prior to detection of the viral RNA in the nucleus 1. Nevertheless, the precise mechanism(s) of IFITM3‐mediated restriction remains unclear. Based on lipid mixing assays, Desai *et al.* suggest that IFITM3 inhibits IAV genome release from endosomes after the formation of hemifusion intermediates 4. While Li et al. suggest that IFITM proteins inhibit the initial lipid mixing events leading to hemifusion, based on cell–cell fusion experiments with IAV and the retrovirus JSRV 3. Our experiments with SFV indicate that virus delivery to acidic endosomes and the initial steps in the fusion reaction, including low pH‐induced conformational changes in the viral envelope protein are unaffected by IFITM3. Nevertheless, delivery of the capsid to the cytosol is blocked, suggesting that IFITM3 inhibits membrane fusion or, possibly, uncoating after fusion has occurred.

It is interesting to note that IFITM1 did not inhibit endocytic entry of either alphavirus since many other viruses show at least some sensitivity to this protein 22. Furthermore, dengue virus, a type II fusion protein expressing virus, similar to alphaviruses, is restricted by IFITM1, 2 and 3 17. We speculate endocytic uptake and fusion with early endosomes allows SFV to escape inhibition from the plasma membrane localized IFITM1, in this system. However, why the endocytic route of alphavirus entry is not inhibited by IFITM1, while other endocytosed, low pH‐dependent viruses (such as dengue) are inhibited, remains unclear.

Previously the effect of IFITM protein expression on SFV E1/E2‐mediated fusion from within (cell–cell fusion facilitated by E1/E2 expressed on the cell surface and exposure of cells to low pH medium) was reported 3. In that study IFITM1 and IFITM3 were found to inhibit syncytium formation. In agreement, we also see that both IFITM1 and IFITM3 can inhibit infection by SFV, when virus is fused at the cell surface. Immunofluorescence (Figure 3 and 20), EM (data not shown) and flow cytometry (Figure 8B) show lower levels of IFITM3 at the cell surface than IFITM1. It is therefore interesting that IFITM3 seems to be able to inhibit infection by cell surface fusion to an equivalent or greater extent than IFITM1 (Figure 8A). Furthermore, when IFITM3 is relocated to the plasma membrane (IFITM3‐Y20A) greater inhibition of infection was seen compared to IFITM1 (Figure 8A). We conclude that IFITM3 has greater potency for inhibiting SFV fusion than IFITM1. Understanding this difference may provide insights to the mechanism(s) underlying the ability of IFITMs to restrict viral replication.

Finally, we observed that the antiviral action of IFITM3 can be saturated by increasing the amount of virus, and is also influenced by the level of IFITM protein expression (Figures 1 and 2). Type I interferon treatment of A549 cells results in similar levels of IFITM expression as seen in the P1 and P2 cell sets (data not shown). This ability to saturate IFITM3 restriction could be explained if there is a level of direct interaction between IFITM3 and SFV fusion sites. Whether IFITM3 is recruited to sites of membrane fusion, or is localized to specific membrane domains conducive for fusion, remains to be established.

With the wealth of experimental data on SFV membrane fusion, using alphaviruses as a model may aid our understanding of the molecular mechanism(s) underlying IFITM‐mediated inhibition of viral infection. Elucidating these mechanisms may help to generate novel therapeutic strategies that could be applied against a broad range of both human and animal pathogens, and to further our understanding of membrane fusion, a phenomenon that underpins many more biological functions than viral entry.

## Materials and Methods

### Cell lines and viruses

The previously described 19, 20 original set (designated OS) of A549 cells stably expressing C‐terminal HA‐tagged human IFITM1, 2 or 3, were cultured in Ham's F‐12 GlutaMAX media (all cell culture reagents were from Life Technologies, unless otherwise stated), supplemented with 10% (v/v) foetal calf serum [FCS (PAA)] and 1% (v/v) Penicillin/Streptomycin (Pen/Strep, 10 000 units/mL, 10 000 µg/mL), as previously described 20. The puromycin 1 (P1) set of cells, which consists of A549 cells stably expressing an empty puromycin resistance vector or C‐terminal HA‐tagged IFITM1, 2 or 3, and the puromycin 2 (P2) set of cells, which consists of A549 cells stably expressing GFP or C‐terminal HA‐tagged IFITM3 or IFITM3‐Y20A, were cultured in the same conditions. BHK‐21 cells were cultured in Glasgow‐MEM supplemented with 5% FCS, 1% Pen/Strep and 10% (v/v) tryptose phosphate broth (Sigma).

SFV stocks were prepared as described 8. Briefly, BHK‐21 cells were infected with SFV at an MOI of 0.05 pfu/cell and cultured for 22 h (h). Supernatants were collected and cleared of cellular debris by low speed centrifugation and the virus concentrated by ultracentrifugation (100,000 × g for 2.5 h at 4 °C). Virus pellets were resuspended in TN buffer (100 mm NaCl, 50 mm Tris pH 7.6) and stored at −80°C. Virus infectivity was determined on BHK‐21 cells by serial dilution plaque assay.

Sindbis virus (SINV) AR339 (a kind gift from Dr. Penny Powell, University of East Anglia, Norwich, UK) stocks were prepared as for SFV.

### Antibodies

Rabbit sera against the SFV envelope glycoprotein (E1/E2) and the capsid protein were previously described 31. Rabbit anti‐SINV sera was provided by Dr. Penny Powell (University of East Anglia, Norwich, UK). Rat anti‐HA (clone 3 F10, Roche), mouse anti‐HA (clone HA.11 16B12, Covance), rabbit anti‐IFITM1‐N‐terminal‐domain (NTD), rabbit anti‐IFITM3‐NTD, mouse anti‐tubulin, goat anti‐rabbit AlexaFluor (AF)488, goat anti‐rat AF647, goat anti‐rabbit IRDye 680 and goat anti‐mouse IRDye 800 were all previously described 20. Mouse anti‐transferrin receptor (TfR, 1 mg/mL, clone MEM‐189, Abcam), mouse anti‐EEA1 (250 µg/mL, BD Biosciences), mouse anti‐CD63 32, goat anti‐rabbit AF594 and goat anti‐mouse AF647 (both 2 mg/mL, Life Technologies). All antibodies were diluted as described below.

### Viral infections

To infect A549 cells, virus was diluted to the required MOI in F‐12 infection media [F‐12 media supplemented with 0.2% (w/v) bovine serum albumin (BSA) and 10 mm HEPES (both Sigma), pH 6.8], and incubated with cells for 5.5–6 h at 37 °C. Infected cells were detected by immunofluorescence staining, as described below, using antibodies against the viral envelope glycoproteins. An Opera confocal microscope (Perkin‐Elmer) was used to image cells and the number of infected cells counted using the columbus software (Perkin‐Elmer).

### Virus binding to the cell surface

SFV particles were bound to the cell surface to promote synchronous internalization in the indicated experiments. Virus was diluted in 4 °C binding media [BM: RPMI medium without bicarbonate (Sigma), supplemented with 0.2% BSA, 10 mm HEPES and 10 mm MES (Sigma) at pH 6.8] and added to cells at the indicated MOIs for 1 h with gentle shaking. The cells were then rinsed twice with cold BM to remove unbound virus, prior to any further treatment, as detailed below.

### SFV internalization

SFV internalization into cells was analyzed by immunofluorescence and EM (described below) or biochemically. For light microscopy analysis, SFV was bound to cells at an MOI of 50 or 200 pfu/cell prior to treatment with pre‐warmed pH 6.8 BM and incubation at 37°C for the indicated times. Cells were then fixed, permeabilized and stained for E1/E2 or the capsid protein.

For the biochemical investigation of internalization, SFV (200 pfu/cell) was bound to cells at 4°C. The cells were then washed and warmed for the indicated times, or left on ice. Surface‐bound virus was removed by treatment with subtilisin [2 mg/mL in PBS (Sigma)] for 1 h at 4°C with gentle shaking. Subtilisin was inactivated by addition of 1 mm PMSF in PBS with 30 mg/mL BSA. Cells were collected and washed with PBS containing 0.2% (w/v) BSA to remove detached virus. Internalized virus was then measured by western blot analysis of SFV E1/E2 in whole cell lysates. Subsequently, the band intensities were quantified: A measure of total cell‐associated virus was determined by averaging the E1 or E2 band intensities at each time point, without subtilisin treatment. The E1 or E2 intensity at each subtilisin treated time point was then set as a proportion of this averaged total, thus giving a percentage of subtilisin resistant (internal) E1 or E2. All intensity values were adjusted based on the tubulin loading control. These values were calculated and averaged over 3–4 experiments.

### SFV endosomal penetration

To determine the kinetics of endosomal penetration in A549 cells, 5 pfu/cell SFV was added and allowed to bind. Subsequently, F‐12 infection media, pre‐warmed to 37°C, containing DMSO or 10 µm monensin, was added to cells (*t* = 0). At indicated time points between 3 and 30 min, DMSO media was replaced with F‐12 infection media containing 10 µm monensin. Cells were then incubated at 37°C for 5.5–6 h, after which they were fixed and analyzed for infection by immunofluorescence microscopy for the E1/E2 proteins.

### Immunofluorescence staining and microscopy and flow cytometry

Immunofluorescence staining and microscopy was performed as previously described 20 with the exception that 0.1% Triton‐X100 (Tx100, Sigma) was used for permeabilization in experiments to detect the SFV capsid protein.

For flow cytometry, cells were detached from plates using 5 mm EDTA and fixed in suspension with 3% formaldehyde for 15 min at room temperature. Formaldehyde was quenched with 50 mm NH_4_Cl in 0.2% BSA diluted in PBS (PBS/BSA) for 15 min at room temperature. Samples were then permeabilized with 0.05% saponin in PBS/BSA [permeabilization buffer (PB)] or incubated with PBS/BSA alone for 30 min at room temperature, prior to labelling with rat anti‐HA antibody for 1 h at room temperature (either in PB or PBS/BSA as appropriate). Cells were washed 3× with the appropriate solution and labeled with goat anti‐rat AF647 for 45 min at room temperature. As controls, samples were incubated with no antibodies, or incubated with only AF647. Cells were washed 3× with PBS and subject to flow cytometry (LSR‐II; BD Bioscience). Cells were gated on forward and side scatter and analyzed for fluorescence intensity. Data were processed using flowjo (v10.1r5) software (Tree Star).

Antibodies were used at the following pre‐determined dilutions form stocks (listed above): anti‐HA 1:100, anti‐IFITM1‐NTD 1:200, anti‐IFITM3‐NTD 1:200, anti‐SFV E1/E2 1:500, anti‐SFV capsid 1:500, anti‐SINV E1/E2 1:500, anti‐TfR 1:200, anti‐EEA1 1:200, anti‐CD63 1:10 000 and anti‐LAMP1 1:500. All secondary antibodies were used at 1:500.

### Image analysis

Image analysis of colocalization between IFITM‐HA and cellular markers was performed using imagej software as previously described 20. For the analysis of SFV E1/E2 colocalization with EEA1 or IFITM3‐HA this procedure was slightly altered. Confocal sections were acquired and the multi‐channel images of E1/E2 (green) co‐stained with HA or EEA1 (magenta) were split into the component channels. These images were processed with an ‘AND’ function to generate a new image containing only pixels that are both green AND magenta (‘overlapping pixels’). A threshold was set to remove background fluorescence, and the area of the remaining pixels was quantified. These ‘overlapping pixels’ were removed from the E1/E2 image, a threshold set, and the area of the remaining ‘green pixels’ was quantified. Relative pixel areas were then used to calculate the ratio between ‘overlapping pixels’ and ‘green pixels’ to determine the level of overlap between E1/E2 and the cellular proteins.

To quantify the SFV capsid cytosolic fluorescence, confocal sections were collected as displayed in Figure 7A and analyzed using imagej software as follows: The channels containing the nuclei and capsid staining were separated. Nuclei were segmented and any signal in the capsid channel that fell within the nuclei area was assumed to be non‐specific and removed from the image. A threshold was then applied to these capsid images to only detect virus puncta, which were similarly removed, and the remaining signal was deemed to be cytosolic. Individual cells were then segmented and the mean fluorescence in each cell calculated.

Significance testing was performed using unpaired student's *T*‐tests and graphpad prism software to compare the mean values of overlapping pixels for colocalization or the difference between mean capsid cytosolic fluorescence.

### Electron microscopy

Epon section EM: 1000 pfu/cell SFV was added to cells grown on coverslips and allowed to bind as above. Cells were subsequently washed and warmed to allow internalization for the indicated times. The coverslips were fixed in EM‐grade 2% paraformaldehyde/2% glutaraldehyde (TAAB Laboratories Equipment, Ltd.) in 0.1 m sodium cacodylate, secondarily fixed for 1 h in 1% osmium tetraoxide/1.5% potassium ferricyanide at 4°C and then treated with 1% tannic acid in 0.1 m sodium cacodylate for 45 min at room temperature. Samples were then dehydrated in sequentially increasing concentration of ethanol solutions, and embedded in Epon resin. Coverslips were inverted onto prepolymerized Epon stubs and polymerized by baking at 60°C overnight. The 70 nm thin sections were cut with a Diatome 45° diamond knife using an ultramicrotome (UC7; Leica). Sections were collected on 1 × 2 mm formvar‐coated slot grids and stained with Reynolds lead citrate.

Immunolabelling EM: 5000 pfu/cell SFV was added to cells and allowed to bind and internalize as above. As previously described 20, cells were fixed with EM‐grade 4% (w/v) paraformaldehyde in 0.1 m phosphate buffer pH 7.4, infused with 2.3 m sucrose, supported in 12% (w/v) gelatin and frozen in liquid nitrogen. Ultrathin (70 nm) cryosections were cut at −120°C and picked up in 1:1 2.3 m sucrose: 2% methylcellulose. Sections were labeled with primary antibody (mouse anti‐HA 1:400), followed by rabbit anti‐mouse intermediate antibody (1:180, DAKO) and protein A‐gold. For double labelling experiments, sections were treated with 1% glutaraldehyde in PBS after the first protein A‐gold incubation and quenched in 15 mm glycine before repeating the single labelling procedure with the second primary antibody (anti‐E1/E2) and a different sized protein A‐gold, as described 33. Finally, sections were contrast stained in 1:9 solution of 4% uranyl acetate: 2% methylcellulose solution pH 4.0. All samples were imaged using a transmission electron microscope (Tecnai T12; FEI) equipped with a charge‐coupled device camera (SIS Morada; Olympus).

Antibodies were used at the following pre‐determined dilutions from stocks (listed above): SFV E1/E2 1:50, mouse anti‐HA 1:400.

### SFV plasma membrane fusion

Cells, in a 96 well plate format, were pre‐incubated with either DMSO or 100 nM bafilomycin A1 (Baf A) in BM for 15 min at 37°C. Cells were then placed on ice and washed with cold BM containing DMSO or Baf A prior to addition of 5 pfu/cell SFV to bind to the cell surface (in the presence of Baf A or DMSO). The cells were washed to remove unbound virus and treated with 37°C BM (containing DMSO or Baf A) adjusted to pH 5.5 for 3 min (or pH 6.8 as control), to trigger viral fusion at the cell surface. Cells were subsequently incubated for 5.5–6 h in F‐12 infection media containing DMSO or Baf A. Control samples, pH 6.8 DMSO, were treated with F12 infection media containing DMSO, allowing viral entry through the normal endosomal route. Cells pre‐treated with Baf A and pH 6.8 media were incubated with infection media containing Baf A, allowing endocytosis but inhibiting low pH‐induced viral fusion in endosomes. Cells pre‐treated with Baf A and given a pH 5.5 pulse were incubated with infection media containing Baf A to trigger fusion at the plasma membrane, while inhibiting acidification of endosomes, such that the low pH trigger was only received at the plasma membrane. Cells pre‐treated with DMSO and given a pH 5.5 pulse were incubated with infection media containing Baf A, to again block endosomal acidification and as a control for Baf A pre‐treatment. Infected cells were detected by immunofluorescence, as described above, using antibodies against E1/E2. Three random epifluorescence images, at 20× magnification, were taken for each well and analyzed for infection percentage using imagej software. Briefly, nuclei were segmented to detect the total number of cells per field. The segmented nuclei were super‐imposed on the fluorescence channel marking infected cells (E1/E2 stained cells) and a ring of 10 pixels was expanded around each super‐imposed nuclei. The E1/E2 fluorescence intensity within this ring was used to score cells as infected or uninfected. The percentage of infected cells was determined for each image and used to determine the percentage of infected cells per well. All infections were carried out in triplicate wells, and the mean infection percentage calculated. In order to test statistical significance of inhibition all infection percentages were set relative to the A549 pH 6.8 DMSO condition within each cell line set (such that OS‐IFITMs were only compared to OS‐A549, for example), thus giving a measure of the relative infection percentage induced by the pH 5.5 treatments. The difference in relative infection percentages was then analyzed across experiments using unpaired student's *T*‐tests in graphpad prism software to compare the difference in relative infection percentage from pH 5.5 treatments in A549 controls against IFITM expressing cells.

### SFV E1 trypsin insensitivity

Cells were initially pre‐incubated with either DMSO or 100 nM Baf A in BM as described for SFV plasma membrane fusion. Virus was added at an MOI of 200 pfu/cell. Where indicated, cells with bound virus were warmed to 37°C to allow virus uptake (with Baf A as labeled), briefly (3 min) treated with pH 5.5 BM, or left on ice. Cells were lysed in 60 μL 1% Tx100 in PBS for 15 min on ice, and the nuclei removed by centrifugation. 40 μL of cell lysate were divided in two. One sample was mixed with 20 μL trypsin [800 µg/mL in 1% Tx100 (Sigma)] and incubated for 10 min at 37 °C, the other with 20 μL 1% Tx100. Both samples were then mixed with 20 μL of soybean trypsin inhibitor [2 mg/mL (Sigma)]. Finally, 6× non‐reducing Laemmli sample buffer (LSB) was added and an equal volume of each sample was separated by SDS‐PAGE and western blotted for SFV E1/E2, as described below. Samples were not heated prior to SDS‐PAGE to maintain the E1 homotrimer.

### Western blotting

Unless otherwise indicated, cell lysates were produced by incubating cells with Tx100 lysis buffer [1% (v/v) Tx100, 150 mm NaCl, 50 mm Tris–HCl at pH 8.0] containing 1× complete protease inhibitor cocktail (Roche) for 15 min on ice. Nuclei were removed by centrifugation and the protein concentrations determined using the bicinochoninic acid method (Thermo Scientific). Equal amounts of protein for each sample were mixed with 3× LSB containing 100 mm dithiothreitol (unless otherwise indicated). Samples were separated on 10% or 15% SDS‐PAGE gels, transferred to PVDF membranes (Immobilon‐FL, Millipore), and blocked with 5% (w/v) milk powder (Marvel) in Tris‐buffered saline (pH 7.4) with 0.05% Tween‐20 (TBST) for 1 h at room temperature. The membranes were incubated with primary antibodies overnight at 4 °C, washed with TBST and probed with appropriate secondary antibodies conjugated to Li‐COR IRDye fluorophores at room temperature before imaging on a Li‐COR Odyssey system. Quantification of band fluorescence intensity was performed using the odyssey software.

Antibodies were used at the following pre‐determined dilutions from stocks (listed above): anti‐SFV E1/E2 1:1000, anti‐IFITM1‐NTD 1:1000, anti‐IFITM3‐NTD 1:500, anti‐VDAC 1:300, anti‐tubulin 1:1000. Both Li‐COR secondary antibodies were used at 1:10 000.

## Supporting information

Editorial Process

**Figure S1: IFITM3 Y20A localizes to the plasma membrane.** P2‐IFITM3‐HA (wild type; WT) and P2‐IFITM3‐Y20A‐HA cells were fixed, permeabilized and labeled with anti‐IFITM1‐NTD antibodies (which cross‐react with IFITM3) followed by AF488 (green). The images were captured using an epifluorescence microscope. WT IFITM3 was seen in intracellular compartments (also see Figure 3), where as IFITM3‐Y20A‐HA was seen at the plasma membrane. Nuclei were detected with Hoechst staining. Scale bar represents 15 µm. Figure S1 – Associated with Figures 2 and 8. The figure displays the localization of the IFITM3‐Y20A mutant, compared to wild type. This localization has been published by others, and is included here as a demonstration of the plasma membrane localization of the mutant in this system.
**Figure S2: Internalized SFV colocalizes with EEA1.** A) SFV (50 pfu/cell) was bound to A549 cells for 1 h at 4°C prior to warming for the indicated periods to promote endocytic uptake. Cells were then fixed and labeled for SFV E1/E2 and EEA1, and visualized with AF488 (green, E1/E2) and AF647 (magenta, EEA1). Single confocal sections are displayed. As seen in Figure 5, E1/E2 labelling at 0 and 5 min was seen as small puncta. At later time points following endocytosis, larger and brighter puncta were seen. EEA1 and E1/E2 were seen to overlap from 10 min, indicating trafficking of SFV to early endosomes. The apparent increase in EEA1 intensity with time was also seen in mock‐infected samples (data not shown), and may be due to cooling and warming the cells. Nuclei were detected with Hoechst staining. Scale bar represents 15 µm. B) The overlap between green (SFV E1/E2) and magenta (EEA1) pixels was quantified over multiple experiments (see Materials and Methods). A total of three independent experiments were performed, and six images taken at 63× magnification. The average ratio of the relative area of overlapping pixels (green and magenta) to green pixels from each experiment is plotted, with the standard deviation used for the error bars. Figure S2 – Associated with Figure 5. This figure is equivalent to the data in Figure 5, but is performed in the control A549 cells, stained for EEA1 and SFV, rather than staining for IFITM3‐HA as in Figure 5.
**Figure S3: EM imaging of SFV uptake.** SFV (1000 pfu/cell) was bound to A549, or OS‐IFITM3‐HA expressing cells for 1 h at 4°C prior to warming for the indicated periods to promote endocytic uptake. Samples were fixed and processed for Epon section EM, as detailed in Materials and Methods. Virus particles were seen at the plasma membrane at 0 min, then in coated vesicles after 5 min at 37°C. By 20 and 30 min, virus particles appear in endosomal structures, but it was hard to distinguish viral particles from other intraluminal vesicles. Figure S3 – Associated with Figure 5. This figure displays Epon EM micrographs for SFV internalization to complement the IF data of Figure 5.
**Figure S4: Immuno‐gold labelling of cryosections and EM imaging of SFV uptake.** SFV (5000 pfu/cell) was bound to cells and allowed to internalize, prior to processing for cryosectioning and immunogold labelling. A) Sections were labeled with antibodies against SFV E1/E2. Viral particles were detected at the cell surface at 0 min. By 30 min viral particles were found within multivesicular bodies in both A549 and OS‐IFITM3‐HA expressing cells. B) Sections were labeled for SFV E1/E2 and the HA‐tag. The primary antibodies were detected with 10 nm colloidal gold (SFV) or 15 nm colloidal gold (HA) conjugated secondary antibodies. There was minimal HA background detected in the A549 cells, whereas most HA labelling in the IFITM3‐HA cells was associated with multivesicular bodies, where SFV particles were detected following 30 min at 37°C. Scale bars represent 200 nm. Figure S4 – Associated with Figure 5. This figure displays immuno‐gold labeled cryosections and EM micrographs for SFV internalization to complement the IF data of Figure 5
**Figure S5: Kinetics of SFV penetration into A549 cells.** SFV (5 pfu/cell) was bound to A549 cells for 1 h at 4°C prior to warming to 37°C with media containing DMSO or 10 µm monensin to allow endocytic uptake. At time points between 3 and 30 min, DMSO containing media was replaced with media containing monensin. After 5.5–6 h infection, the cells were fixed and analyzed for infection by immunofluorescence microscopy. The data show the percentage of infected cells compared to DMSO controls. Although monensin added at early time points effectively inhibited infection, addition at 30 min had almost no effect. The data displayed are mean infection percentage from three independent infections (each containing duplicates of each sample) with standard deviation between experiments as error bars. Figure S5 – Associated with Figures 4 and 5, 6 and 7. This figure details the results for the monensin time of addition experiment to determine the time course for SFV passing the pH‐dependent step of entry.
