# Supplementary material for: Alphavirus Restriction by IFITM Proteins
Source: Traffic. 2016 Jun 24;17(9):997–1013. doi: 10.1111/tra.12416 (PMC5025721; doi:10.1111/tra.12416)
Supplement: Supplementary file 1 — Editorial Process [file TRA-17-997-s001.docx]

| Title: | **Alphavirus restriction by IFITM proteins** |
| --- | --- |
| Authors: | Stuart Weston, Stephanie Czieso, Ian J. White, Sarah E. Smith, Rachael S. Wash, Carmen Diaz-Soria, Paul Kellam and Mark Marsh |
| Article Type: | Original Research |
|  |  |
| Monitoring Editor | Michael Marks |
| Date Submitted | 16 February 2016 |
| Date for Decision 1 | 10 March 2016 |
| Date Resubmitted | 6 May 2016 |
| Accepted | 6 May 2016 |

**Decision and Reviews**

Dear Mark,

Thank you for submitting your manuscript “Alphavirus restriction by IFITM proteins” to Traffic. I asked three colleagues (sorry, should have been just 2...but as you'll see, #3 was very gentle!) who are experts in the field to review the paper and their verbatim comments are appended below. I share the enthusiasm of the referees for the high caliber of the work presented in this paper and agree that this work will be of interest to the readers of Traffic. Referee 1 and 2 have recommended including additional data to clarify and statistically validate the data shown, and I would encourage you to do this if possible. All three authors raise additional concerns that should be easily addressed by revision of the text and by additional quantification of existing data.

Although I cannot accept your manuscript for publication at this point, I believe that you will be able to address the referees’ concerns relatively easily and I look forward to receiving your revised manuscript. To expedite handling when you resubmit please be sure to include a response outlining how you have addressed each of the referees’ concerns.

Best,

Mickey

Michael S. Marks, Ph.D.
Co-Editor
________________________________________________________

Referee's Comments to the Authors

Referee: 1

Comments to the Author
In this manuscript, Weston et al. study the inhibition of the alphaviruses Sindbis virus and Semliki Forest by the IFITM proteins 1-3. These membrane proteins are induced by IFN and have been shown to inhibit the entry of a number of viruses including alphaviruses, but the precise mechanism is not well-understood. The manuscript reports that IFITM 3 is the most efficient inhibitor, while IFITM 1 has no effect. A step-wise dissection of the various steps of the SFV entry pathway is performed to determine the stage at which inhibition takes place. The authors conclude that IFITM3 inhibits the release of SFV capsid from the endosome into the cytosol. Interestingly, relocalization of IFITM 3 to the PM allows inhibition of fusion at the PM. The work is carefully performed and documented. Although the results do not solve the mechanism by which these proteins inhibit, the study does provide important information on the inhibition and specificity towards this medically important set of viruses, and will be of interest to the field.
The following points should be addressed:

Major points:
1. Previous studies, including the Li et al. paper using SFV E1/E2 in cell-cell fusion, have suggested that IFITM proteins inhibit virus infection by inhibiting the hemifusion step between the virus and cell membrane. However, this step in alphavirus entry is currently overlooked in this manuscript. As there are hemifusion assays available that can be used with alphaviruses in cells, such as dequenching of DiD labeled virus, addition of hemifusion studies would significantly add to this manuscript.
2. Several points about the E1 protein need to be clarified. The high molecular weight band is presumably the homotrimer, since it is trypsin resistant, but many studies in the literature show that the homotrimer is resistant to SDS at 37 but not at higher temperatures. Why is the homotrimer not dissociated in this experiment—how was solubilization carried out? Also, the authors somewhat over-interpret their results (p8) to suggest that the E1 protein not only forms the trimer but is inserted into the endosome membrane. In fact, the virus E1 forms a homotrimer without any association with membranes or cholesterol, so it is premature to conclude that fusion is blocked after membrane insertion.
3. For Fig. 8, IFITM restriction of virus entry is made at the cell surface. In order for the authors to make the comparisons between cell expression and restriction that are in the manuscript and to validate the conclusion that IFITM3 is a more potent inhibitor, it is necessary to quantitatively determine the level of IFITM expression on the cell surface (by FACS or similar assay) rather than extrapolating from total cell expression data as these numbers may not correlate directly.
4. The authors argue that the disappearance of punctate capsid and appearance of diffuse capsid staining represents release of capsid into the cytoplasm. Please show a quantitation of the capsid puncta from a number of cells for control vs. monensin vs. IFITM3 cells. Clarify in the text that the assay is not influenced by capsid biosynthesis—was this tested?
5. Please mention or show the quantitation (%) for the SFV colocalization with IFITM3 (Fig. 5) and EEA1 (Fig. S3).
6. It seems an overstatement to suggest that high amounts of virus somehow saturate FITM3. Indeed, addition of large amounts of virus beyond the linear range of infection can overcome inhibition by NH4Cl, for example. Seems more likely to be that inhibition is strong but not 100%, and thus if you add enough virus significant infection will occur.


Minor points:
In figure 5, why is the bound virus not visualized at time 0?
In Fig. S3, why is the IFITM3 in MVB, when the authors are strongly suggesting that it is in early endosomes?


Referee: 2

Comments to the Author
IFITM proteins are host cell anti-viral proteins that target the stage of virus entry. Many questions remain, however, about IFITMs including why certain IFITMs exert stronger effects on different viruses and their precise mechanism(s) of action. Here Weston et al. investigate the role of IFITMs 1, 2, and 3 in restricting two alphaviruses: Sindbis and SFV. They show (main results) that for normal endocytic entry of Sindbis and SFV, (a) IFITM3 is most effective, with partial activity for IFITM2, and no activity for IFITM1 (Figs. 1 and 2) and that (b) IFITM3 blocks a late step of SFV entry (capsid release into the cytoplasm), after transport to endosomes and low pH induced conformational changes in E1 and E2 (Figs. 5-7). In Fig. 8, they provide evidence that IFITM3 is a more potent inhibitor of fusion than IFITM1 (but see major comment 2).
Major Comments
1. Several statements re: IFITM localization (e.g., IFITM3 “is predominantly localized to early endosomes”) should be modified (since considerable amounts of IFITMs 2 and 3 are found outside of either early or late endosomes). For example, it would be more appropriate to say (e.g., near end of Intro.) that higher levels of IFITM3 are found in early vs. late endosomes (and give % values), and vice versa for IFITM2 (and give % values). Regarding the above, the authors should comment on what other authors have concluded about the locations of IFITMs 1, 2 and 3.
2. Several aspects of Fig. 8 should be clarified:
a. Statistics (and % inhibition values) should be provided to rigorously assess if IFITM1 reduce the ability of SFV to fuse at the surface of OS cells in response to a low pH pulse.
b. The inhibitory effect of IFITM1 in this assay seems greater in P1 vs. OS cells. Is this borne out by % inhibition values and statistics? If so, why do the authors think this is?
c. Fig. 8C would be improved by having a direct comparison of the effects of Y20A IFITM3 and IFITM1.
d. Related to the above, the authors should provide data on relative levels of IFITMs 1 and 3 at the respective cell surfaces (by flow cytometry or cell surface biotinylation) for the Fig. 8 experiments.
3. A statement in the Discussion should be changed: “and the interaction of the acidified E1/E2 with cholesterol-containing endosomal membranes [28] are unaffected by IFITM3”. This is a strong inference of the conformational change data provided by the authors, but a direct measurement of target membrane binding is not presented and so the issue of whether IFITMs block the lipid or content mixing stages of fusion (e.g., as in references 3 and 4) remains to be resolved for alpha- and other IFITM-restricted viruses.
Minor Comments
1. Fig. 2 legend: Define A, 1, 2, 3. (presumably parental A549s and A549s expressing IFITMs 1, 2, and 3, respectively)
2. P4, column 1, ~3/4 way down: What is meant by +ve in “IFITM3+ve”?


Referee: 3

Comments to the Author
In this manuscript, the authors describe detailed studies to show that Sindbis Virus and Semliki Forest Virus (SFV), two Alphaviruses, are specifically restricted by IFITM3, to a minor extend by IFITM1, but not by IFITM2. IFITM are interferon-induced transmembrane proteins that confine the infection of many enveloped viruses. Analysing all know intermediate steps to initiate an SFV infection, the authors demonstrate convincingly, that IFITM3 did not interfere with virus binding, virus internalisation and the low pH-induced conformational changes of the SFV fusion proteins suggesting that virus fusion is still initiated despite an interaction with IFITM3. However, the SFV capsids were not released into the cytosol or at least not uncoated and the capsid proteins dispersed within the cytosol, and thus no virus transcription and viral protein synthesis was initiated.
The manuscript is very well written, and the data and the figures clearly support the conclusions that are made. The study provides a major advance in understanding the cell biology mechanisms how IFITM3 limits the infection of enveloped viruses.

Specific Comments:
1. The author use the term “cytoplasm” in their manuscript for what is actually the “cytosol”. The cytoplasm is the sum of the cytosol, the cytoskeleton and the cytoplasmic organelles. Thus a viral capsid internalised into endosomes, and contained within endosomes due to an inhibition of viral fusion is located as much in the cytoplasm as a cytosolic capsid. In other words, a capsid that is released from the virions upon virus fusion at the plasma membrane or an endosomal membrane is released into the cytosol. So in the present study, IFITM3 prevents the dispersal of capsid antigens in the cytosol. Please change the text accordingly wherever this distinction is relevant for the understanding of the cell biological mechanisms involved.
2. This reviewer found the mixed nomenclature between the main text and the labels in the figures regarding the different cell lines used confusing. It would be easier for the readers if the authors could stick to one name, e.g. P1-IFITM2-HA or P1-IFITM2, but not both. The shorter version could be defined in the Materials & Methods section describing the cell lines, and then be used throughout the manuscript.
3. Why does the appearance of the subcellular localization of IFITM3-HA (Fig. 5) and EEA1 (Fig. S3) change during the course of the viral infection? Is this due to the warming-up after virus binding on ice as it would also occur during warming up of mock-infected cells, or is this an effect of virus infection as it would also occur during an unsynchronized infection at 37 °C? Please clarify.
4. Please rearrange the numbering of the supplementary figures; at present Fig. S4 is described before Figure S2.
5. The X axis of Fig. S2 is skewed. Please use an arithmetic presentation, indicating the temporal changes in accordance to the time passed; in other words, the length of the axis representing 0 to 10 min should equal that for 10 to 20 min.
6. Discussion: To my cell biology view of the cell, it seems that IFITM3 might interfere with changes in the actin cytoskeleton required to expand the fusion pores to such an extent that the viral capsids could indeed be released into the cytosol. Is there anything known about such a potential connection? If yes, please integrate this information into the discussion.

**Author Rebuttal**

Dear Mickey,

Thanks for your email of 10th March 2016 containing the reviews of our paper on IFITM restriction of alpha virus infection. We appreciate your and the reviewers’ positive comments and the recommendations for improving the manuscript. We have made significant changes and provide here a point-by-point response to each comment.

We hope you find these modifications appropriate, and that the paper is now acceptable for publication in Traffic.

Best wishes,
Mark

Mark Marsh

Referee: 1

Comments to the Author
In this manuscript, Weston et al. study the inhibition of the alphaviruses Sindbis virus and Semliki Forest by the IFITM proteins 1-3. These membrane proteins are induced by IFN and have been shown to inhibit the entry of a number of viruses including alphaviruses, but the precise mechanism is not well-understood. The manuscript reports that IFITM 3 is the most efficient inhibitor, while IFITM 1 has no effect. A step-wise dissection of the various steps of the SFV entry pathway is performed to determine the stage at which inhibition takes place. The authors conclude that IFITM3 inhibits the release of SFV capsid from the endosome into the cytosol. Interestingly, relocalization of IFITM 3 to the PM allows inhibition of fusion at the PM. The work is carefully performed and documented. Although the results do not solve the mechanism by which these proteins inhibit, the study does provide important information on the inhibition and specificity towards this medically important set of viruses, and will be of interest to the field.
The following points should be addressed:

We thank the reviewer for pointing out that alphaviruses have been suggested to be inhibited by IFITM proteins as we had missed data from a cDNA screen suggesting potential inhibition of Chikungunya virus by IFITMs (though limited inhibition of Venezuelan equine encephalitis virus was seen in that study). We have included mention of this work (Schoggins et al. 2011) in the introduction.

Major points:
1. Previous studies, including the Li et al. paper using SFV E1/E2 in cell-cell fusion, have suggested that IFITM proteins inhibit virus infection by inhibiting the hemifusion step between the virus and cell membrane. However, this step in alphavirus entry is currently overlooked in this manuscript. As there are hemifusion assays available that can be used with alphaviruses in cells, such as dequenching of DiD labeled virus, addition of hemifusion studies would significantly add to this manuscript.

We are aware of the study by Li et al. Given that they studied experimentally induced cell-cell fusion, we wanted to determine the impact of IFITM protein expression on virus infection. As our data show clear differences in the activities of IFITM proteins during virus infection compared to that reported by Li et al. As our results suggest that IFITM3 also inhibits alphavirus fusion we are working towards understanding whether this occurs at the hemifusion step. However, it is our view that this is a study in its own right and, given the substantial amount of data in this paper, inclusion of lipid mixing studies is beyond the scope of the current manuscript and would compromise a subsequent publication.

2. Several points about the E1 protein need to be clarified. The high molecular weight band is presumably the homotrimer, since it is trypsin resistant, but many studies in the literature show that the homotrimer is resistant to SDS at 37 but not at higher temperatures. Why is the homotrimer not dissociated in this experiment—how was solubilization carried out? Also, the authors somewhat over-interpret their results (p8) to suggest that the E1 protein not only forms the trimer but is inserted into the endosome membrane. In fact, the virus E1 forms a homotrimer without any association with membranes or cholesterol, so it is premature to conclude that fusion is blocked after membrane insertion.

We are aware that the homotrimer is sensitive to high temperatures. Thus for these experiments, following trypsin treatment and addition of SBTI, aliquots of the lysates were mixed with 6x Laemmli sample buffer and separated by electrophoresis without heating. This is now stated clearly in the Materials and Methods (page 9). We have changed the text to remove discussion of E1 membrane insertion.

3. For Fig. 8, IFITM restriction of virus entry is made at the cell surface. In order for the authors to make the comparisons between cell expression and restriction that are in the manuscript and to validate the conclusion that IFITM3 is a more potent inhibitor, it is necessary to quantitatively determine the level of IFITM expression on the cell surface (by FACS or similar assay) rather than extrapolating from total cell expression data as these numbers may not correlate directly.

We have previously reported that IFITM3 expression at the plasma membrane is low, when analysed by immunofluorescence (Weston et al. 2014). We have now added FACS data (Fig. 8B) that show low levels of IFITM3 at the cell surface compared to IFITM1 and IFITM3-Y20A.

4. The authors argue that the disappearance of punctate capsid and appearance of diffuse capsid staining represents release of capsid into the cytoplasm. Please show a quantitation of the capsid puncta from a number of cells for control vs. monensin vs. IFITM3 cells. Clarify in the text that the assay is not influenced by capsid biosynthesis—was this tested?

We have now included a quantitative analysis of the capsid release as Fig. 7B and included details in the Materials and Methods (page 8). Briefly, confocal sections were collected, background fluorescence associated with nuclei was removed, along with the punctate capsid staining at the cell surface or within endosomes, leaving only cytosolic fluorescence. The mean fluorescence intensity was then measured within each cell to determine the changes in cytosolic fluorescence over time.

5. Please mention or show the quantitation (%) for the SFV colocalization with IFITM3 (Fig. 5) and EEA1 (Fig. S3).

Fig. 5 and S2 now include graphs displaying quantitative analysis of the overlap between SFV E1/E2 with HA or EEA1 over time. These data were generated by analysing images essentially as for Fig. 3 (detailed in Materials and Methods on page 8).


6. It seems an overstatement to suggest that high amounts of virus somehow saturate IFITM3. Indeed, addition of large amounts of virus beyond the linear range of infection can overcome inhibition by NH4Cl, for example. Seems more likely to be that inhibition is strong but not 100%, and thus if you add enough virus significant infection will occur.

While we accept the point made by the reviewer regarding loss of inhibition from NH4Cl when virus input is increased, we are of the option that since increasing virus input or having lower levels of IFITM expression both reduce the level of inhibition, this may still represent a saturation affect.

Minor points:
In figure 5, why is the bound virus not visualized at time 0?

This was an issue with the brightness and contrast of the original image. A new version is now included in which the levels have been increased equally in all panels. It is still difficult to see the virus particles at early time points, presumably because these are single virions sparsely distributed over the cells. Virus staining becomes more obvious as endocytosis causes virions to cluster in endocytic organelles.

In Fig. S3, why is the IFITM3 in MVB, when the authors are strongly suggesting that it is in early endosomes?

While we see that IFITM3 has most overlap with markers of early endosomes (Fig. 3), the protein is not exclusively within early endosomes, as also displayed in Fig. 3. We are also of an opinion that IFITM3 may stimulate the formation of MVBs, which is the source of further research but outside the scope of the current study.

Referee: 2

Comments to the Author
IFITM proteins are host cell anti-viral proteins that target the stage of virus entry. Many questions remain, however, about IFITMs including why certain IFITMs exert stronger effects on different viruses and their precise mechanism(s) of action. Here Weston et al. investigate the role of IFITMs 1, 2, and 3 in restricting two alphaviruses: Sindbis and SFV. They show (main results) that for normal endocytic entry of Sindbis and SFV, (a) IFITM3 is most effective, with partial activity for IFITM2, and no activity for IFITM1 (Figs. 1 and 2) and that (b) IFITM3 blocks a late step of SFV entry (capsid release into the cytoplasm), after transport to endosomes and low pH induced conformational changes in E1 and E2 (Figs. 5-7). In Fig. 8, they provide evidence that IFITM3 is a more potent inhibitor of fusion than IFITM1 (but see major comment 2).

Major Comments
1. Several statements re: IFITM localization (e.g., IFITM3 “is predominantly localized to early endosomes”) should be modified (since considerable amounts of IFITMs 2 and 3 are found outside of either early or late endosomes). For example, it would be more appropriate to say (e.g., near end of Intro.) that higher levels of IFITM3 are found in early vs. late endosomes (and give % values), and vice versa for IFITM2 (and give % values). Regarding the above, the authors should comment on what other authors have concluded about the locations of IFITMs 1, 2 and 3.

We have included some discussion of IFITM localisations in the text (page 3).


2. Several aspects of Fig. 8 should be clarified:
a. Statistics (and % inhibition values) should be provided to rigorously assess if IFITM1 reduce the ability of SFV to fuse at the surface of OS cells in response to a low pH pulse.
b. The inhibitory effect of IFITM1 in this assay seems greater in P1 vs. OS cells. Is this borne out by % inhibition values and statistics? If so, why do the authors think this is?
c. Fig. 8C would be improved by having a direct comparison of the effects of Y20A IFITM3 and IFITM1.
d. Related to the above, the authors should provide data on relative levels of IFITMs 1 and 3 at the respective cell surfaces (by flow cytometry or cell surface biotinylation) for the Fig. 8 experiments.

A) We have now attempted to analyse the inhibition in a statistical way as detailed in Materials and Methods (page 9). For this, normalised infection values were calculated by comparison to A549 pH 6.8 DMSO within each cell set and then performing student’s T-tests on the normalised infection percentages seen with either of the pH 5.5 treatments.

B) As pointed out by the reviewer, and as can be now seen in the statistical analysis, the inhibitory affect of OS-IFITM1 does appear to be lower than P1-IFITM1. However, this difference appears to be related to differences in the control A549 cells. Both OS-IFITM1 and P1-IFITM1 show similar average infection of 10-15%, following pH 5.5 treatments. By comparison OS-A549 cells show an average infection of 20-23%, and P1-A549 cells an average of 51-52% when similarly treated. The P2-A549 cells show an infection of 27-31%. Thus, it appears there may be differences in the permissiveness of the background cell lines to infection by plasma membrane fusion. The three A549 control lines are different: OS-A549 cells are ‘standard’ A549s (which are parental to all other lines), P1-A549 and P2-A549s were lentivirally transfected with an empty puromycin resistance vector and a GFP vector, respectively. Overall, our interpretation from the two cell groups (OS and P1) is that IFITM1 can inhibit SFV infection by plasma membrane fusion, but that this is inefficient. All IFITM3 cells (OS, PI and P2) show significant differences to their A549 controls, supporting our conclusion that IFITM3 is more potent in inhibiting SFV infection by plasma membrane fusion than IFITM1.

C) No IFITM1 line was produced in the P2 set, so for Y20A and IFITM1 we can only currently compare P2-Y20A with OS-IFITM1 or P1-IFITM1, as displayed.

D) As discussed above, a FACS based analysis of surface levels of each IFITM has now been included as Fig. 8B.

2. A statement in the Discussion should be changed: “and the interaction of the acidified E1/E2 with cholesterol-containing endosomal membranes [28] are unaffected by IFITM3”. This is a strong inference of the conformational change data provided by the authors, but a direct measurement of target membrane binding is not presented and so the issue of whether IFITMs block the lipid or content mixing stages of fusion (e.g., as in references 3 and 4) remains to be resolved for alpha- and other IFITM-restricted viruses.

As indicated above, we have removed this interpretation of the data.

Minor Comments
1. Fig. 2 legend: Define A, 1, 2, 3. (presumably parental A549s and A549s expressing IFITMs 1, 2, and 3, respectively)

The figure legend has been changed accordingly.

2. P4, column 1, ~3/4 way down: What is meant by +ve in “IFITM3+ve”?

Text has been changed to read “IFITM positive endosomes.”


Referee: 3

Comments to the Author
In this manuscript, the authors describe detailed studies to show that Sindbis Virus and Semliki Forest Virus (SFV), two Alphaviruses, are specifically restricted by IFITM3, to a minor extend by IFITM1, but not by IFITM2. IFITM are interferon-induced transmembrane proteins that confine the infection of many enveloped viruses. Analysing all know intermediate steps to initiate an SFV infection, the authors demonstrate convincingly, that IFITM3 did not interfere with virus binding, virus internalisation and the low pH-induced conformational changes of the SFV fusion proteins suggesting that virus fusion is still initiated despite an interaction with IFITM3. However, the SFV capsids were not released into the cytosol or at least not uncoated and the capsid proteins dispersed within the cytosol, and thus no virus transcription and viral protein synthesis was initiated.
The manuscript is very well written, and the data and the figures clearly support the conclusions that are made. The study provides a major advance in understanding the cell biology mechanisms how IFITM3 limits the infection of enveloped viruses.

We thank the reviewer for his/her positive comments.

Specific Comments:
1. The author use the term “cytoplasm” in their manuscript for what is actually the “cytosol”. The cytoplasm is the sum of the cytosol, the cytoskeleton and the cytoplasmic organelles. Thus a viral capsid internalised into endosomes, and contained within endosomes due to an inhibition of viral fusion is located as much in the cytoplasm as a cytosolic capsid. In other words, a capsid that is released from the virions upon virus fusion at the plasma membrane or an endosomal membrane is released into the cytosol. So in the present study, IFITM3 prevents the dispersal of capsid antigens in the cytosol. Please change the text accordingly wherever this distinction is relevant for the understanding of the cell biological mechanisms involved.

We thank the reviewer for pointing out our error – the senior author, at least, should have known better! The text has been corrected.

2. This reviewer found the mixed nomenclature between the main text and the labels in the figures regarding the different cell lines used confusing. It would be easier for the readers if the authors could stick to one name, e.g. P1-IFITM2-HA or P1-IFITM2, but not both. The shorter version could be defined in the Materials & Methods section describing the cell lines, and then be used throughout the manuscript.

We thank the reviewer for pointing out the issue and have altered the nomenclature to improve clarity.


3. Why does the appearance of the subcellular localization of IFITM3-HA (Fig. 5) and EEA1 (Fig. S3) change during the course of the viral infection? Is this due to the warming-up after virus binding on ice as it would also occur during warming up of mock-infected cells, or is this an effect of virus infection as it would also occur during an unsynchronized infection at 37 °C? Please clarify.

The apparent changes do indeed seem to be a result of the cells being placed on ice and then warmed, as similar results were seen in mock-infected cells. This has been mentioned in the manuscript but we have decided not to include the data.


4. Please rearrange the numbering of the supplementary figures; at present Fig. S4 is described before Figure S2.

We thank the reviewer for pointing out this error and have corrected the supplemental figure order accordingly.


5. The X axis of Fig. S2 is skewed. Please use an arithmetic presentation, indicating the temporal changes in accordance to the time passed; in other words, the length of the axis representing 0 to 10 min should equal that for 10 to 20 min.

All graphs involving time have been changed accordingly.


6. Discussion: To my cell biology view of the cell, it seems that IFITM3 might interfere with changes in the actin cytoskeleton required to expand the fusion pores to such an extent that the viral capsids could indeed be released into the cytosol. Is there anything known about such a potential connection? If yes, please integrate this information into the discussion.

To our knowledge there has been no study indicating an association between IFITM3 and cytoskeletal dynamics, however, we thank the reviewer for this interesting idea and will take it into consideration.

**Decision and Reviews**

Dear Mark,

Thank you so much for resubmitting your manuscript, “Alphavirus restriction by IFITM proteins”, to your old pals at Traffic. As all three reviewers suggested only minor changes to the manuscript, I have read your response to the previous reviews and the changes that you have made to the manuscript myself. I am pleased to say that I found your responses to be thorough and appropriate, and with one very small exception, the changes to the manuscript were spot on. The only point I felt that you could have addressed better in the manuscript was your response to Referee 2, question 2b. I agreed with Referee #2 that the difference in the apparent inhibitory effect of IFITM1 between P1 and OS cells was concerning. Your response to the referee makes perfect sense, but I think that other readers might have the same question. I thus suggest that you allude to the differences in the permissiveness of the parent cell lines to pH 5.5 infection in the text of the manuscript. With that change, I will be pleased to accept your manuscript for publication. Thanks so much for sending it in!

Yours,

Mickey

Michael S. Marks, Ph.D.
Co-Editor

_____________________________________________________________________________
