# Supplementary material for: Alphavirus Restriction by IFITM Proteins
Source: Traffic. 2016 Jun 24;17(9):997–1013. doi: 10.1111/tra.12416 (PMC5025721; doi:10.1111/tra.12416)
Supplement: Supplementary file 2 — Figure S1: IFITM3 Y20A localizes to the plasma membrane. P2‐IFITM3‐HA (wild type; WT) and P2‐IFITM3‐Y20A‐HA cells were fixed, permeabilized and labeled with anti‐IFITM1‐NTD antibodies (which cross‐react with IFITM3) followed by AF488 (green). The images were captured using an epifluorescence microscope. WT IFITM3 was seen in intracellular compartments (also see Figure 3), where as IFITM3‐Y20A‐HA was seen at the plasma membrane. Nuclei were detected with Hoechst staining. Scale bar represents 15 µm. Figure S1 – Associated with Figures 2 and 8. The figure displays the localization of the IFITM3‐Y20A mutant, compared to wild type. This localization has been published by others, and is included here as a demonstration of the plasma membrane localization of the mutant in this system. Figure S2: Internalized SFV colocalizes with EEA1. A) SFV (50 pfu/cell) was bound to A549 cells for 1 h at 4°C prior to warming for the indicated periods to promote endocytic uptake. Cells were then fixed and labeled for SFV E1/E2 and EEA1, and visualized with AF488 (green, E1/E2) and AF647 (magenta, EEA1). Single confocal sections are displayed. As seen in Figure 5, E1/E2 labelling at 0 and 5 min was seen as small puncta. At later time points following endocytosis, larger and brighter puncta were seen. EEA1 and E1/E2 were seen to overlap from 10 min, indicating trafficking of SFV to early endosomes. The apparent increase in EEA1 intensity with time was also seen in mock‐infected samples (data not shown), and may be due to cooling and warming the cells. Nuclei were detected with Hoechst staining. Scale bar represents 15 µm. B) The overlap between green (SFV E1/E2) and magenta (EEA1) pixels was quantified over multiple experiments (see Materials and Methods). A total of three independent experiments were performed, and six images taken at 63× magnification. The average ratio of the relative area of overlapping pixels (green and magenta) to green pixels from each experiment is plotted, with th [file TRA-17-997-s002.docx]

**Alphavirus restriction by IFITM proteins**

Stuart Weston^a^, Stephanie Czieso^a^, Ian J. White^a^, Sarah E. Smith^b^, Rachael S. Wash^b^, Carmen Diaz-Soria^b^, Paul Kellam^b,c^ and Mark Marsh^a,*^

^a^MRC Laboratory for Molecular Cell Biology, University College London, Gower Street, London, WC1E 6BT, UK

^b^ Wellcome Trust Sanger Institute, Wellcome Trust Genome Campus, Hinxton, CB10 1SA, UK

^c^ Division of Infection and Immunity, University College London, Gower Street, London, WC1E 6BT, UK

*Email: [m.marsh@ucl.ac.uk](mailto:m.marsh@ucl.ac.uk)

Telephone ++44 (0) 20 7679 7807

**Supplemental Material**


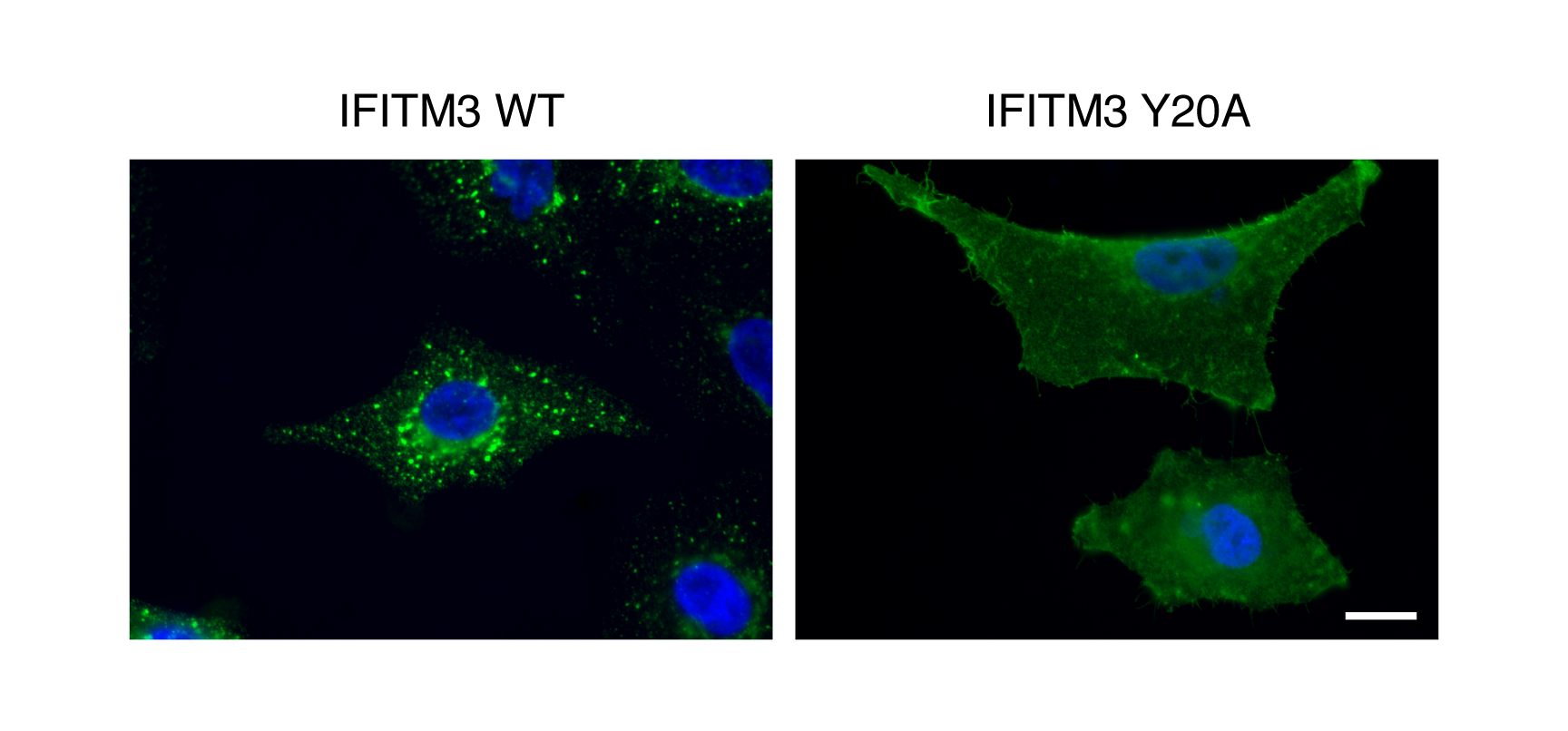


**Supplemental Figure 1. IFITM3 Y20A localises to the plasma membrane**

P2-IFITM3-HA (wild type; WT) and P2-IFITM3-Y20A-HA cells were fixed, permeabilised and labelled with anti-IFITM1-NTD antibodies (which cross-react with IFITM3) followed by AF488 (green). The images were captured using an epifluorescence microscope. WT IFITM3 was seen in intracellular compartments (also see Fig. 3), where as IFITM3-Y20A-HA was seen at the plasma membrane. Nuclei were detected with Hoechst staining. Scale bar represents 15 μm.


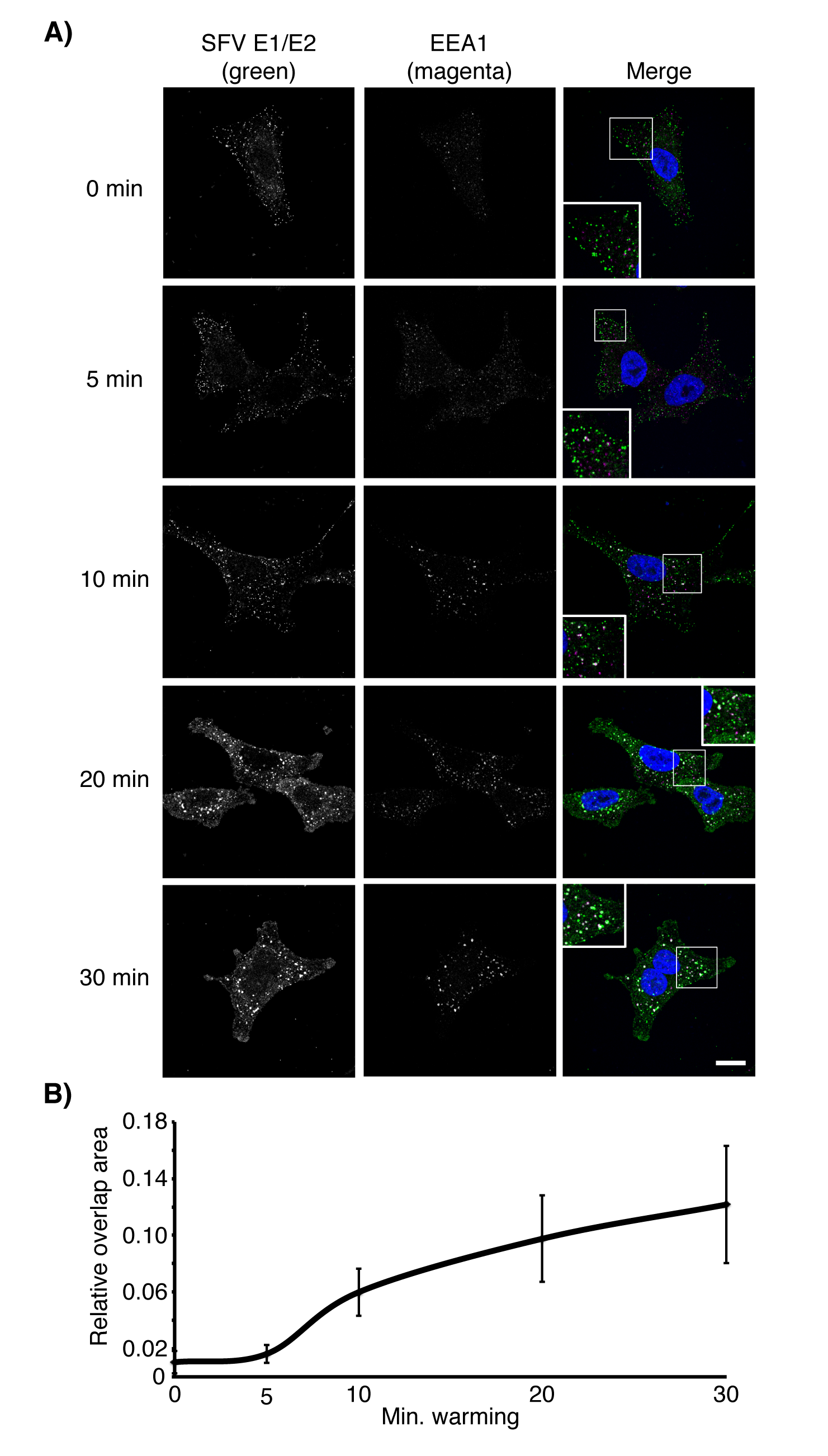


**Supplemental Figure 2. Internalised SFV co-localises with EEA1**

A) SFV (50 pfu/cell) was bound to A549 cells for 1 h at 4°C prior to warming for the indicated periods to promote endocytic uptake. Cells were then fixed and labelled for SFV E1/E2 and EEA1, and visualised with AF488 (green, E1/E2) and AF647 (magenta, EEA1). Single confocal sections are displayed. As seen in Fig. 5, E1/E2 labelling at 0 and 5 min was seen as small puncta. At later time points following endocytosis, larger and brighter puncta were seen. EEA1 and E1/E2 were seen to overlap from 10 min, indicating trafficking of SFV to early endosomes. The apparent increase in EEA1 intensity with time was also seen in mock infected samples (data not shown), and may be due to cooling and warming the cells. Nuclei were detected with Hoechst staining. Scale bar represents 15 μm. B) The overlap between green (SFV E1/E2) and magenta (EEA1) pixels was quantified over multiple experiments (see Materials and Methods). A total of 3 independent experiments were performed, and 6 images taken at 63x magnification. The average ratio of the relative area of overlapping pixels (green and magenta) to green pixels from each experiment is plotted, with the standard deviation used for the error bars.

**
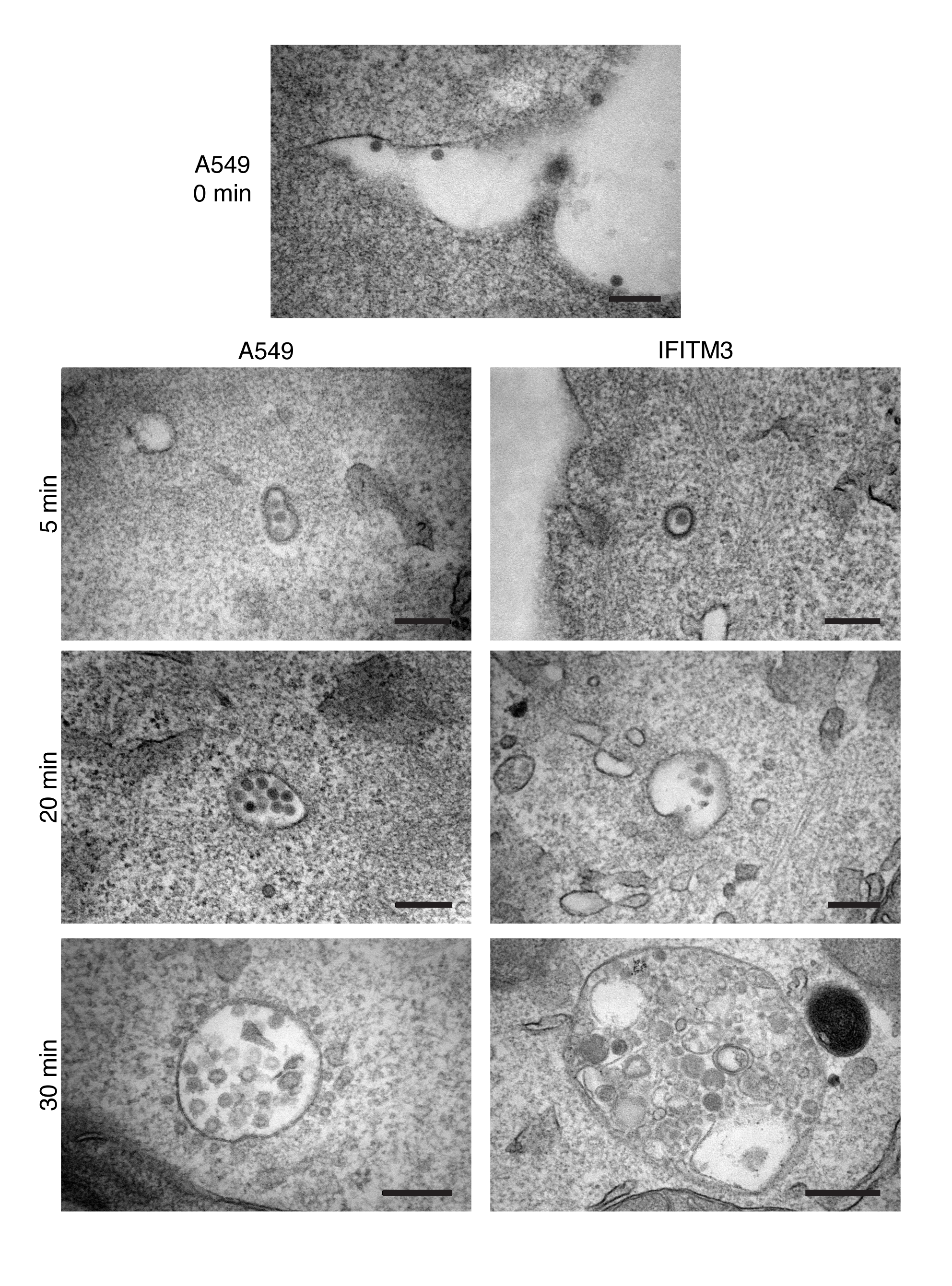
**

**Supplemental Figure 3. EM imaging of SFV uptake**

SFV (1000 pfu/cell) was bound to A549, or OS-IFITM3-HA expressing cells for 1 h at 4°C prior to warming for the indicated periods to promote endocytic uptake. Samples were fixed and processed for Epon section EM, as detailed in Materials and Methods. Virus particles were seen at the plasma membrane at 0 min, then in coated vesicles after 5 min at 37°C. By 20 and 30 min, virus particles appear in endosomal structures, but it was hard to distinguish viral particles from other intraluminal vesicles.


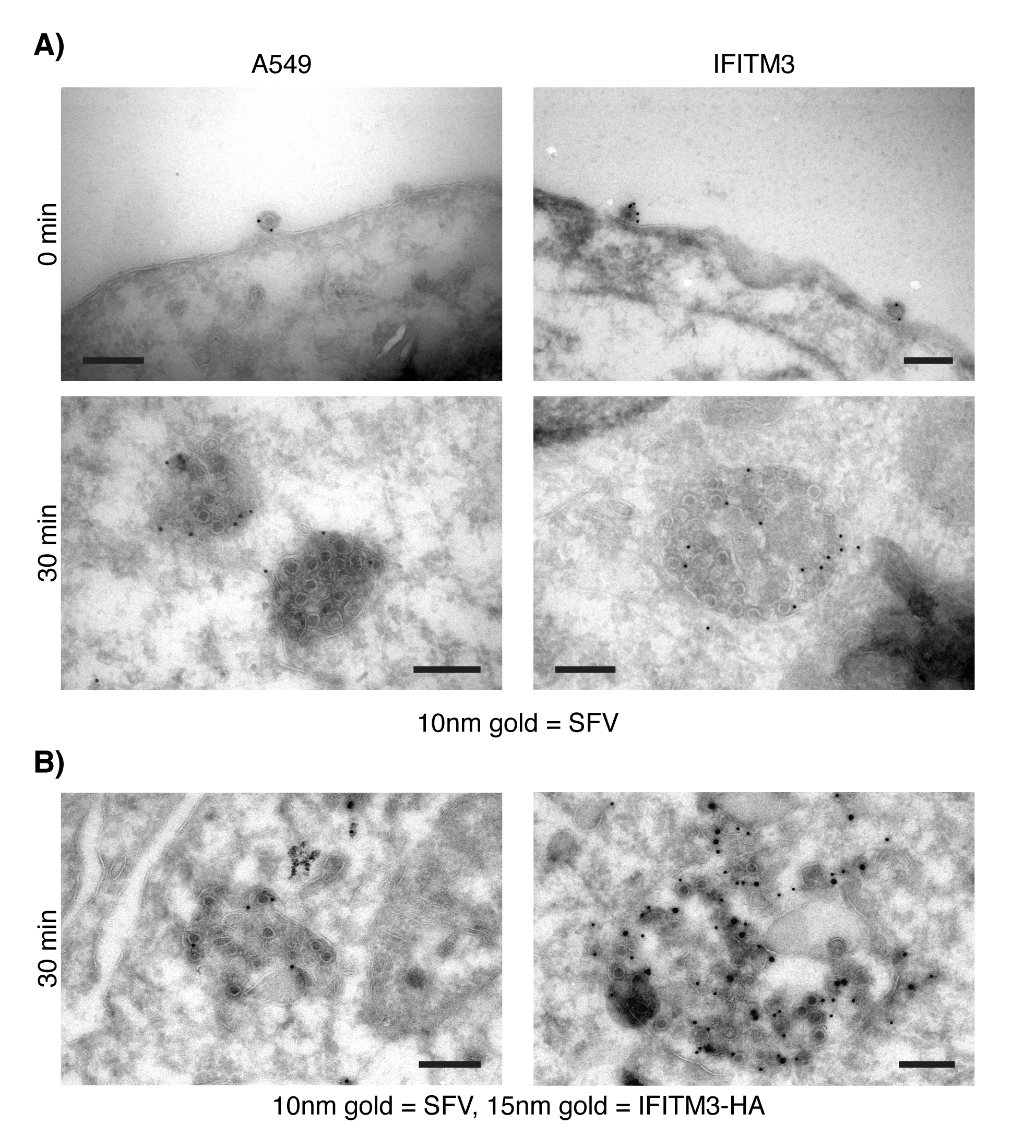


**Supplemental Figure 4. Immuno-gold labelling of cryosections and EM imaging of SFV uptake**

SFV (5000 pfu/cell) was bound to cells and allowed to internalise, prior to processing for cryosectioning and immunogold labelling. A) Sections were labelled with antibodies against SFV E1/E2. Viral particles were detected at the cell surface at 0 min. By 30 min viral particles were found within multivesicular bodies in both A549 and OS-IFITM3-HA expressing cells. B) Sections were labelled for SFV E1/E2 and the HA-tag. The primary antibodies were detected with 10 nm colloidal gold (SFV) or 15 nm colloidal gold (HA) conjugated secondary antibodies. There was minimal HA background detected in the A549 cells, whereas most HA labelling in the IFITM3-HA cells was associated with multivesicular bodies, where SFV particles were detected following 30 min at 37°C. Scale bars represent 200 nm.

**
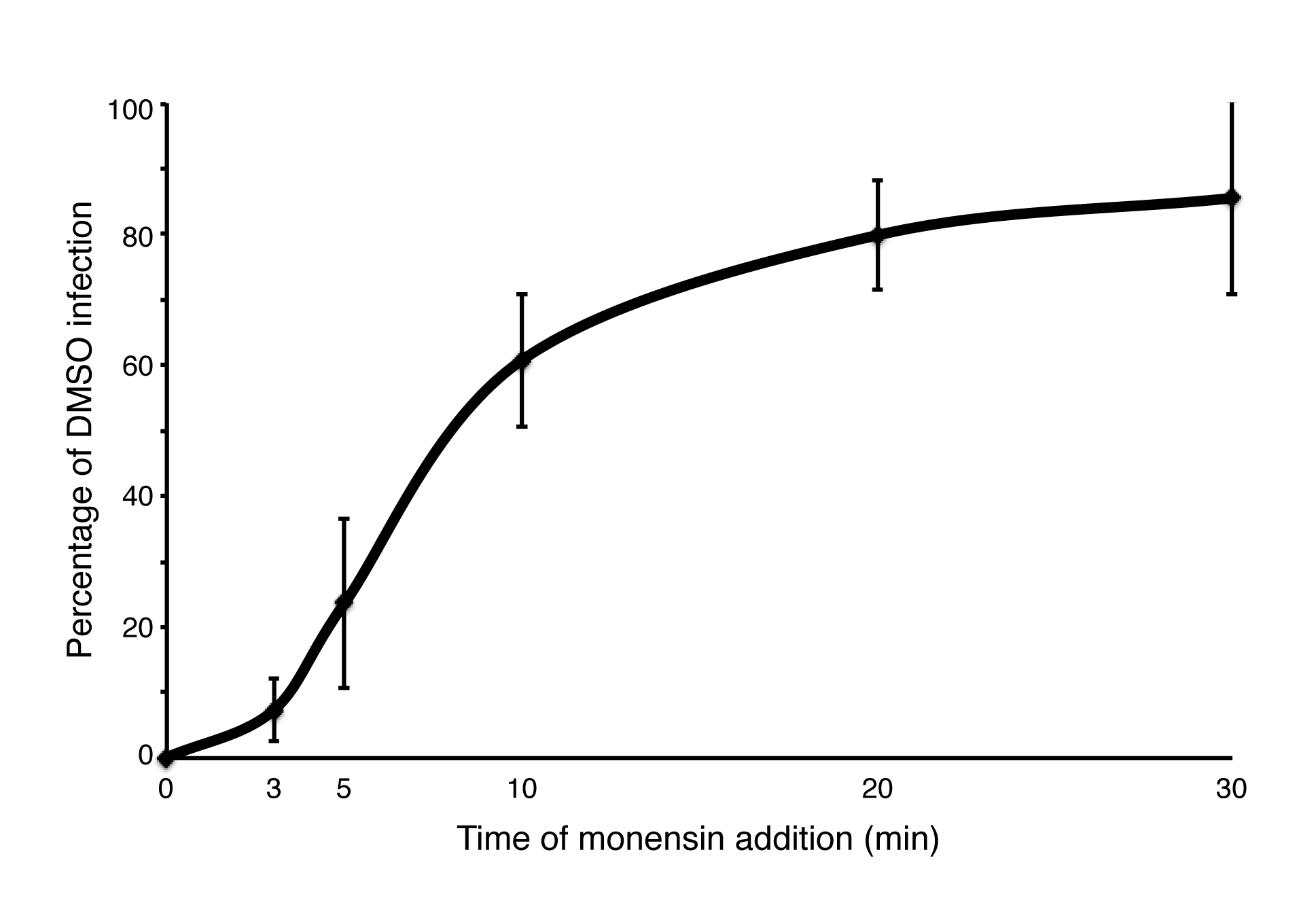
**

**Supplemental Figure 5. Kinetics of SFV penetration into A549 cells.**

SFV (5 pfu/cell) was bound to A549 cells for 1h at 4°C prior to warming to 37°C with media containing DMSO or 10 μM monensin to allow endocytic uptake. At time points between 3 and 30 min, DMSO containing media was replaced with media containing monensin. After 5.5-6 h infection, the cells were fixed and analysed for infection by immunofluorescence microscopy. The data show the percentage of infected cells compared to DMSO controls. Although monensin added at early time points effectively inhibited infection, addition at 30 min had almost no effect. The data displayed are mean infection percentage from 3 independent infections (each containing duplicates of each sample) with standard deviation between experiments as error bars.
